# Global prevalence and biopsychosocial correlates of psychological distress among people living with HIV: an updated theory-informed meta-analysis

**DOI:** 10.3389/fpubh.2026.1832557

**Published:** 2026-06-19

**Authors:** Qiaoting Xue, Qilian He, Zheng Zhu, Hongli Yang, Xin He

**Affiliations:** 1 School of Nursing, Dali University, Dali, China; 2 School of Nursing, Fudan University, Shanghai, China; 3 Yunnan Provincial Hospital of Infectious Diseases, Kunming, China; 4 Nanchong Central Hospital, Nanchong, China

**Keywords:** associated factors, meta-analysis, people living with HIV, prevalence, psychological distress, systematic review

## Abstract

**Introduction:**

Psychological distress (PD) is common among people living with HIV (PLWH), but prevalence estimates vary substantially. This study aimed to synthesize the prevalence of PD among PLWH and examine its heterogeneity and associated factors.

**Methods:**

Following PRISMA 2020, we systematically searched seven electronic databases from inception to June 20, 2025, for observational studies on PD prevalence and associated factors among PLWH. PD was operationalized as a binary outcome using validated self-report instruments with explicit cut-offs. The pooled prevalence was estimated using a generalized linear mixed model, and associated factors were synthesized as pooled ORs. Subgroup analyses and univariable meta-regression were performed to explore heterogeneity. Study quality was assessed, and the certainty of evidence was rated using the Grading of Recommendations Assessment, Development and Evaluation framework.

**Results:**

Sixteen studies (10,204 PLWH) were included. The pooled prevalence of PD was 0.40 (95% CI: 0.28–0.53), with considerable heterogeneity (*I^2^* = 96.25%). Sensitivity analysis yielded a broadly similar estimate. Meta-regression suggested that geographic region and PD tool category were associated with prevalence heterogeneity. Poor ART adherence (OR = 4.55, 95% CI: 1.96–10.54), non-disclosure of HIV status (OR = 4.95, 95% CI: 3.23–7.58), low CD4 count (OR = 2.59, 95% CI: 1.62–4.13), and female sex (OR = 2.26, 95% CI: 1.67–3.07) were associated with higher odds of PD, whereas being married was associated with lower odds of PD (OR = 0.35, 95% CI: 0.26–0.47).

**Conclusion:**

PD is a common burden among PLWH, and this review provides an updated evidence base to identify vulnerable populations, refine measurement, and inform care priorities, though findings should be interpreted cautiously due to considerable heterogeneity and low to very low certainty of evidence.

**Systematic review registration:**

https://www.crd.york.ac.uk/PROSPERO/view/CRD420251089847, identifier (CRD420251089847).

## 1 Introduction

The Human Immunodeficiency Virus (HIV) and its resultant Acquired Immunodeficiency Syndrome (AIDS) remain a significant global public health challenge. According to the 2025 Global AIDS Update report released by The Joint United Nations Programme on HIV/AIDS, an estimated 40.8 million people were living with HIV/AIDS (PLWH) globally by the end of 2024, with 1.3 million new infections and approximately 630,000 AIDS-related deaths recorded in 2024 alone (1). Antiretroviral therapy (ART) has been scaled up to cover 31.6 million people (1), transforming HIV into a manageable chronic condition (2). As survival has improved and HIV care has increasingly shifted toward long-term disease management, the psychosocial and mental health burden experienced by PLWH has become more visible in clinical and public health practice. This burden includes psychological distress, depressive and anxiety symptoms, stigma-related stress, and difficulties related to disclosure, social relationships, and sustained engagement in care (3, 4).

In AIDS psychological research, psychological distress (PD) is increasingly recognized as a clinically significant construct (5). While distinct from diagnosable anxiety and depressive disorders, it is frequently conflated with these conditions (6). For PLWH, PD represents a broader, non-specific, and multidimensional construct (7, 8). HIV-related PD has been conceptualized as an actual or potential multidimensional painful experience arising from intertwined physiological, psychological, social, and spiritual impairments or trauma, encompassing four core dimensions: emotional distress, illness-coping distress, identity distress, and social interaction distress (9). However, existing quantitative research has been hampered by conceptual ambiguity. Most studies have employed generic PD measurement tools or, more problematically, used anxiety or depression symptomatology as proxy measures for PD (10–13). This approach risks failing to capture the multidimensional suffering unique to PLWH, particularly overlooking individuals who experience significant distress without meeting conventional diagnostic thresholds, thereby constraining the development of targeted early identification and intervention strategies (9).

To systematically investigate this complex phenomenon, we integrate two complementary theoretical frameworks, the Biopsychosocial Model (BPS) and Lazarus’s Transactional Model of Stress and Coping (TMSC). The BPS provides a structural scaffold to categorize potential determinants of PD across biological, psychological, and social domains (14). The TMSC supplies the dynamic psychological sequence of “stress-appraisal-coping-outcome” (15), theorizing that PD arises when PLWH appraise HIV-related stressors as exceeding their coping resources, leading to maladaptive coping efforts and significant PD (16, 17). Together, this integrated framework provides a theory-informed structure for organizing associated factors and interpreting how biological, psychological/behavioral, and social conditions may be linked to PD among PLWH.

To address the identified gaps, this review delivers three advancements over the prior synthesis (18), which was limited by its outdated evidence (cutoff 2019) and lack of meta-analysis on associated factors. First, it provides a timeliness update by synthesizing evidence up to 2025. Second, it introduces the BPS-TMSC framework to systematically synthesize associated factors. Third, it responds to the WHO mhGAP initiative’s call to prioritize mental health care for PLWH by providing context-sensitive implications for practice, measurement, and future research (19). The specific aims are to: (1) estimate the updated global prevalence of PD among PLWH; and (2) identify and quantify its associated factors within the BPS-TMSC framework.

## 2 Methods

### 2.1 Protocol and registration

This systematic review was conducted in accordance with the Joanna Briggs Institute (JBI) Manual for Evidence Synthesis (20) and reported following the Preferred Reporting Items for Systematic Reviews and Meta-Analyses (PRISMA) 2020 statement (See Supplementary material S1) (21). The protocol was prospectively registered with PROSPERO (CRD420251089847). As this study synthesized data from previously published research, ethical approval was not required.

### 2.2 Search strategy

We systematically searched PubMed, Web of Science, Embase, Cochrane Library, CINAHL, PsycInfo, and CNKI from database inception to June 20, 2025. No language restriction was applied during the database search. Records published in languages other than English or Chinese were first screened using available English titles or abstracts; if a potentially eligible study was identified, translation would be used to assess the full text and extract data where necessary. The final included studies were published in English or Chinese. The search strategy combined controlled vocabulary (e.g., MeSH terms) with free-text terms related to “HIV/AIDS” and “psychological distress” (e.g., “HIV,” “emotional distress”), and was adapted for each database. Full search strategies for each database are provided in Supplementary material S2. To ensure comprehensive coverage, we also manually screened the reference lists of all included articles.

### 2.3 Operational definition of PD

In this review, PD was conceptualized as a broad, non-specific, and clinically meaningful state of emotional suffering among PLWH, rather than as a synonym for any single psychiatric disorder. For study eligibility, PD had to be operationalized as a binary outcome using a validated self-report instrument with an explicit cutoff value. Because no universally adopted HIV-specific PD instrument has been consistently used across countries and care settings, we adopted a hierarchical operational strategy. Eligible instruments included HIV-specific PD tools, general distress tools, and symptom-oriented tools when they captured clinically relevant distress or distress-related symptoms and provided a threshold for case identification. Anxiety or depression-oriented instruments, such as HADS or PHQ-4, were therefore treated as symptom-oriented proxy indicators of PD rather than as equivalent representations of the full PD construct. When more than one eligible PD measure was reported within the same study, one estimate was selected according to a pre-specified hierarchy: HIV-specific PD instruments, followed by general distress instruments and then symptom-oriented tools. If multiple eligible instruments were available within the same category, we selected the measure used as the primary outcome in the original article; if no primary measure was specified, we selected the instrument most consistent with the broad operational definition of PD used in this review. Only one prevalence estimate per study was included in the primary pooled analysis to avoid double-counting. Potential heterogeneity related to measurement approach was further examined through subgroup analysis and meta-regression.

### 2.4 Inclusion and exclusion criteria

Studies were included if they: (1) enrolled people living with HIV/AIDS; (2) reported the prevalence of PD and associated factors; (3) measured PD in accordance with our pre-specified operational definition; (4) provided sufficient data to calculate effect estimates (e.g., prevalence with sample size, odds ratios (ORs) with 95% confidence intervals (CIs), or data to compute them); and (5) used an observational design (cross-sectional, cohort, or case–control).

Studies were excluded if they: (1) did not assess PD as an outcome; (2) were duplicate publications or used overlapping datasets (for overlapping datasets, we retained the publication with the largest sample size or most comprehensive analysis); (3) were non-original research (e.g., reviews, conference abstracts, theses, case reports); or (4) had no full text available.

### 2.5 Study selection and data extraction

All identified records were imported into EndNote X9, and duplicates were removed through automatic and manual checking. Two reviewers (XQT and HX) independently screened titles and abstracts against the eligibility criteria, then retrieved and reviewed the full texts of potentially eligible studies. Disagreements were resolved through discussion or by arbitration of a third reviewer (HQL).

Data extraction was performed independently by the same two reviewers using a pre-piloted data extraction form. The following information was extracted: (1) study characteristics, including first author, publication year, country, and study design; (2) participant characteristics, including population type, sample size, gender distribution, and age; (3) PD measurement characteristics, including the specific tool(s) used, cutoff threshold for defining PD, and the conceptual definition of PD as provided by the original authors; when multiple eligible tools were reported, information on all tools was extracted, but only one prevalence estimate was selected for the primary pooled analysis according to the hierarchy described above; and (4) outcome data, including prevalence of PD and adjusted odds ratios (AORs) with 95% confidence intervals (CIs) for associated factors, with the most fully adjusted estimates preferentially extracted.

### 2.6 Risk of bias

Two reviewers (XQT and HX) independently assessed the methodological quality of the included studies. Disagreements were resolved by consensus or by a third reviewer (HQL). For cross-sectional studies, we used the Agency for Healthcare Research and Quality (AHRQ) tool (22), which comprises 11 items scored as 1 (“yes”) or 0 (“no”/“unclear”). Studies were categorized as high (score ≥8), moderate (4–7), or low (≤3) quality. For cohort and case–control studies, we applied the Newcastle-Ottawa Scale (NOS) (23, 24), which assesses selection, comparability, and outcome/exposure across nine stars. Studies were rated as high (7–9 stars), moderate (5–6 stars), or low (0–4 stars) quality.

### 2.7 Certainty of evidence

We assessed the certainty of evidence for each pooled outcome using the Grading of Recommendations Assessment, Development and Evaluation (GRADE) framework across five domains: risk of bias, inconsistency, indirectness, imprecision, and publication bias.

### 2.8 Data synthesis and analysis

All analyses were performed using Stata 18.0. Pooled prevalence was calculated using a generalized linear mixed model (GLMM) with a logit link. For associated factors, pooled AORs were selected per study. All ORs were log-transformed and synthesized using random-effects models with the Hartung-Knapp-Sidik-Jonkman (HKSJ) adjustment. For narrative presentation, study-reported associated factors were organized according to the BPS framework into biological, psychological/behavioral, and social or contextual domains. Heterogeneity was quantified by the *I^2^* statistic and tau-squared (*τ^2^*), estimated via restricted maximum likelihood (REML). Pre-specified subgroup analyses and meta-regression were conducted to explore potential sources of heterogeneity. Study-level variables included: study period (pre 2015: early chronic HIV care with limited mental health integration; 2015–2019: mature long-term management; post 2019: additional COVID-19 impacts); geographic region (Sub-Saharan Africa, Europe/North America, and Asia); PD tool category (general distress tools: GHQ-12, SRQ-20, K-10; HIV-specific: HRPDS; symptom tools: HADS, PHQ-4, BSI, IDPSQ-14, IES-R); and population type (general PLWH and special subpopulations). Economic region and COVID-19 period were additionally examined as supplementary exploratory subgroup variables. Sensitivity analyses included leave-one-out and HKSJ random-effects analysis on logit-transformed estimates. Given the small number of studies and sparse categories, analyses of study-level characteristics associated with heterogeneity were interpreted as exploratory. Publication bias was assessed using Egger’s test, with Begg’s test and funnel plots additionally used only when the number of included studies for a given pooled analysis was at least 10 (k ≥ 10, where k denotes the number of studies).

## 3 Results

### 3.1 Study selection

**Figure 1:**
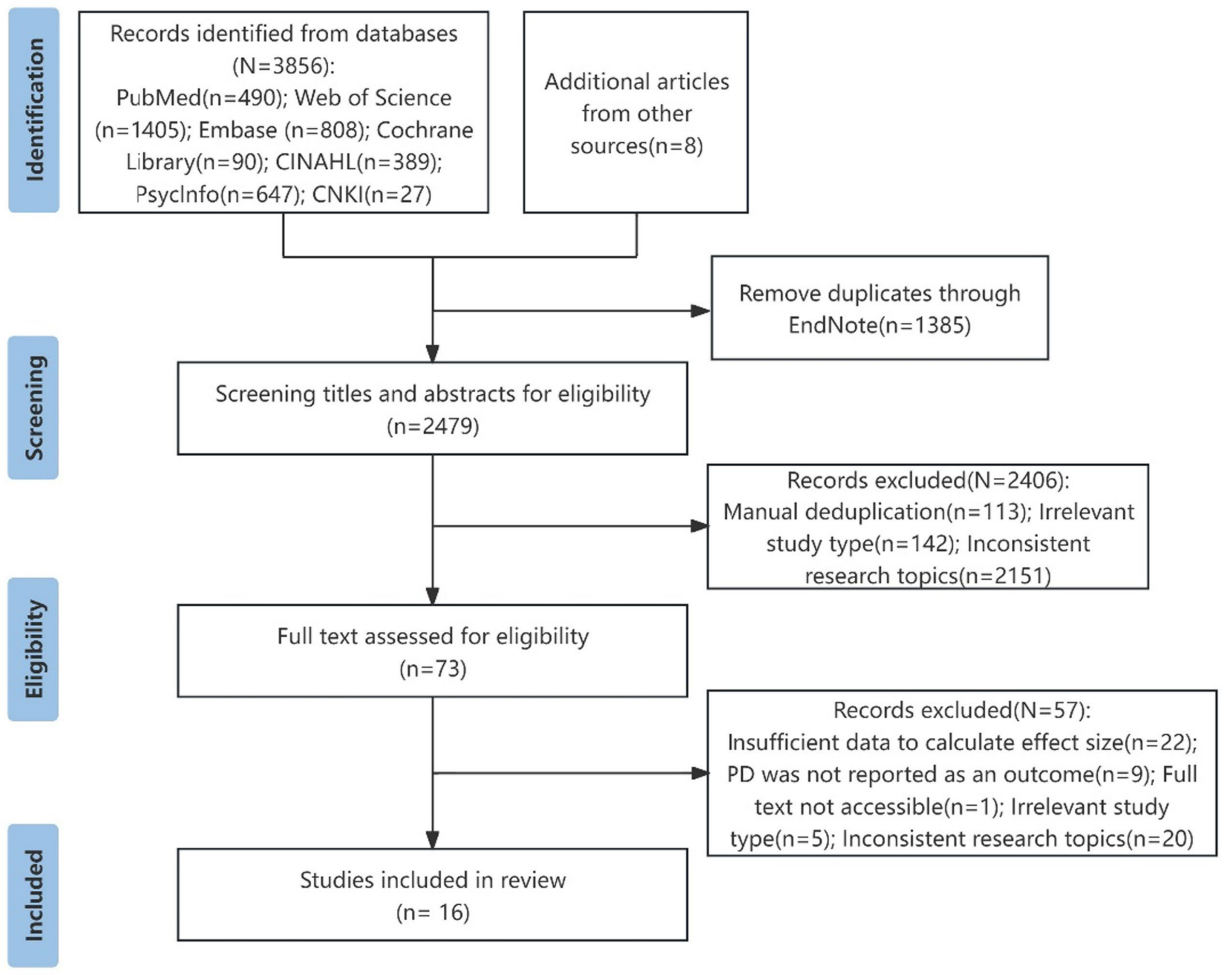
PRISMA flowchart of study selection process for a meta-analysis of PD among PLWH.

The systematic search retrieved 3,856 records, with an additional eight records identified through screening the reference lists of eligible articles. After removing 1,385 duplicates using EndNote, 2,479 records remained. Following title and abstract screening, 2,406 records were excluded, including 113 duplicates removed manually. The full texts of the remaining 73 articles were assessed for eligibility. Of these, 57 were excluded based on the predefined criteria, leaving 16 studies that met the inclusion criteria and were included in the systematic review and meta-analysis (11–13, 25–37), (Figure 1).

### 3.2 Characteristics of included studies

This review synthesized 16 studies involving 10,204 PLWH. A total of 3,472 individuals were identified with PD, with prevalence estimates ranging widely from 7.8 to 83.3%. The studies were published between 2007 and 2024 and were conducted across multiple settings, including Sub-Saharan Africa, Europe/North America, and Asia. According to World Bank classifications (38), seven studies were conducted in lower-middle-income or low-income countries. Detailed characteristics are presented in Table 1.

**Table 1:** Characteristics and risk of bias of included studies.

Study	Country	Study design	Population	Female (%)	Age (years)	n of PLWH	Prevalence of PD (%)	Screening tool for PD	Risk of bias
Akoko et al. (11)	Nigeria	CO	PLWH	62.0%	40.04 ± 9.21	288	37.0% (31.4–42.6)	SRQ-20 (≥5/20)	7 (High)
Vondo et al. (13)	South Africa	CS	PLWH	68.9%	N/A	4,401	27.4% (25.3–29.7)	K-10 (≥20/50)	7 (Moderate)
Kesande et al. (31)	Uganda	CS	Youth living with HIV	100.0%	21.1 ± 2.40	224	40.2% (33.8–46.6)	K-10 (≥20/50)	8 (High)
Ma et al. (32)	China	CS	PLWH	32.8%	29.6 ± 6.7	765	83.3% (80.6–85.9)	HRPDS (≥63/110)	7 (Moderate)
Pierce et al. (35)	Nigeria	CO	PLWH	62.0%	38(33–46)	485	33.8% (29.7–38.2)	SRQ-20 (≥5/20)	8 (High)
Brouillette et al. (12)	Canada	CO	PLWH	10.4%	57.3 ± 7.0	77	32.5% (22.1–44.7)	HADS (subscale ≥11/21)	6 (Moderate)
Solomon et al. (36)	United Kingdom	CS	Older women living with HIV	100.0%	N/A	724	27.1% (23.9–30.3)	PHQ-4 (≥6/12)	7 (Moderate)
Donne et al. (29)	Italy	CS	PLWH	24.5%	N/A	98	45.0% (35.0–55.0)	IES-R (≥24/88)	5 (Moderate)
Moges et al. (33)	Ethiopia	CS	Newly diagnosed PLWH	57.8%	33 ± 9.17	689	58.6% (55.2–62.3)	K-10 (≥20/50)	8 (High)
Garriga (30)	Spain	CS	PLWH	17.6%	Female:40 ± 10 male:38 ± 10	563	40.9% (36.8–45.0)	GHQ-12 (≥3/12)	8 (High)
Basha et al. (25)	Ethiopia	CS	PLWH	62.8%	39.15 ± 10.44	422	7.8% (5.3–10.4)	SRQ-20 (≥11/20)	7 (Moderate)
Monteiro et al. (34)	Portugal	CS	Middle-aged/older women living with HIV	76.4%	N/A	508	15.0% (12.0–18.0)	BSI (≥63)	7 (Moderate)
Blais et al. (28)	Canada	CS	Mothers living with HIV	100.0%	40.8 ± 6.6	100	45.0% (35.3–54.7)	IDPSQ-14 (≥26.19/56)	6 (Moderate)
Benoit et al. (26)	Canada	CS	Women and trans women living with HIV	100.0%	43(35–50)	337	42.4% (37.1–47.7)	K-10 (≥20/50)	9 (High)
Tesfaye and Bune (37)	Ethiopia	CS	PLWH	58.0%	34.8 ± 8.92	500	11.2% (8.4–13.9)	HADS (total ≥19/42)	7 (Moderate)
Bernatsky et al. (27)	Angola	CC	Pregnant women living with HIV	100.0%	23.8	23	66.7% (47.7–85.7)	GHQ-12 (≥3/12)	5 (Moderate)

CO, Cohort; CS, Cross-sectional; CC, Case–control; PLWH, People living with HIV/AIDS; Youth living with HIV, aged 15–24 years; Older women living with HIV, age 45–60 years; Newly diagnosed PLWH, within 30 days of diagnosis; SRQ-20, 20-item self-reporting questionnaire; K-10, Kessler psychological distress scale; HRPDS, HIV-related psychological distress scale; HADS, Hospital anxiety and depression scale; PHQ-4, Patient health questionnaire-4; IES-R, Impact of event scale-revised; GHQ-12, 12-item general health questionnaire; BSI, brief symptom inventory; IDPSQ-14, Indice de détresse psychologique de Santé Québec-14; N/A, Not reported.

### 3.3 Variation in conceptualizing and measuring PD

Substantial heterogeneity was observed in how PD was conceptualized and measured across the included studies. Conceptually, most studies (10/16) did not provide an independent theoretical definition of PD and instead relied on the scoring criteria of the measurement tools. Among those that offered explicit descriptions, PD was variably framed as a broad emotional state (25, 30) or represented indirectly through anxiety or depression-related symptom measures (26, 35, 37). Methodologically, the instruments used to identify PD differed considerably in their underlying construct coverage and cutoff thresholds. GHQ-12, SRQ-20, and K-10 were grouped as general distress tools because they assess broad emotional or psychological distress. HADS, PHQ-4, BSI, IDPSQ-14, and IES-R were grouped as symptom tools because they primarily assess anxiety, depressive, trauma-related, or other symptom-oriented manifestations. HRPDS was treated as the HIV-specific PD instrument because it captures HIV-related distress dimensions, including illness coping, identity, disclosure, and social interaction distress. These measurement differences indicate that the included tools captured partially overlapping but non-equivalent dimensions of PD. Therefore, variation in prevalence estimates may reflect differences in the distress constructs assessed by each instrument, in addition to differences in study population, setting, and cutoff thresholds.

### 3.4 Risk of bias

The risk of bias is presented in Table 1, with detailed criteria and ratings provided in Supplementary material S3. Overall, the included studies demonstrated moderate quality with specific methodological limitations. Among the 12 cross-sectional studies, only half clearly specified whether subjects were consecutively enrolled (26, 31, 33, 34, 36, 37); all failed to describe quality assurance procedures and follow-up completeness; and only two adequately explained the handling of missing data (26, 30). Among the four cohort and case–control studies, all failed to provide a clear definition of control groups, and only one reported non-response rates (35).

### 3.5 Certainty of evidence

The certainty of evidence was assessed using GRADE (see Supplementary material S4). The initial certainty for all evidence was low. The assessment revealed that no evidence was rated as high or moderate certainty. The prevalence estimate was rated very low, mainly due to serious inconsistency and imprecision. Certainty for all associated factors was low or very low; risk of bias was the common reason for downgrading, with inconsistency and imprecision further limiting the evidence for ART adherence, and sparse data for CD4 count. Although some factors (e.g., female sex, being married) showed large effects, this did not warrant upgrading.

### 3.6 Meta-analysis of prevalence and associated factors

#### 3.6.1 Pooled prevalence and sensitivity analysis of PD

**Figure 2:**
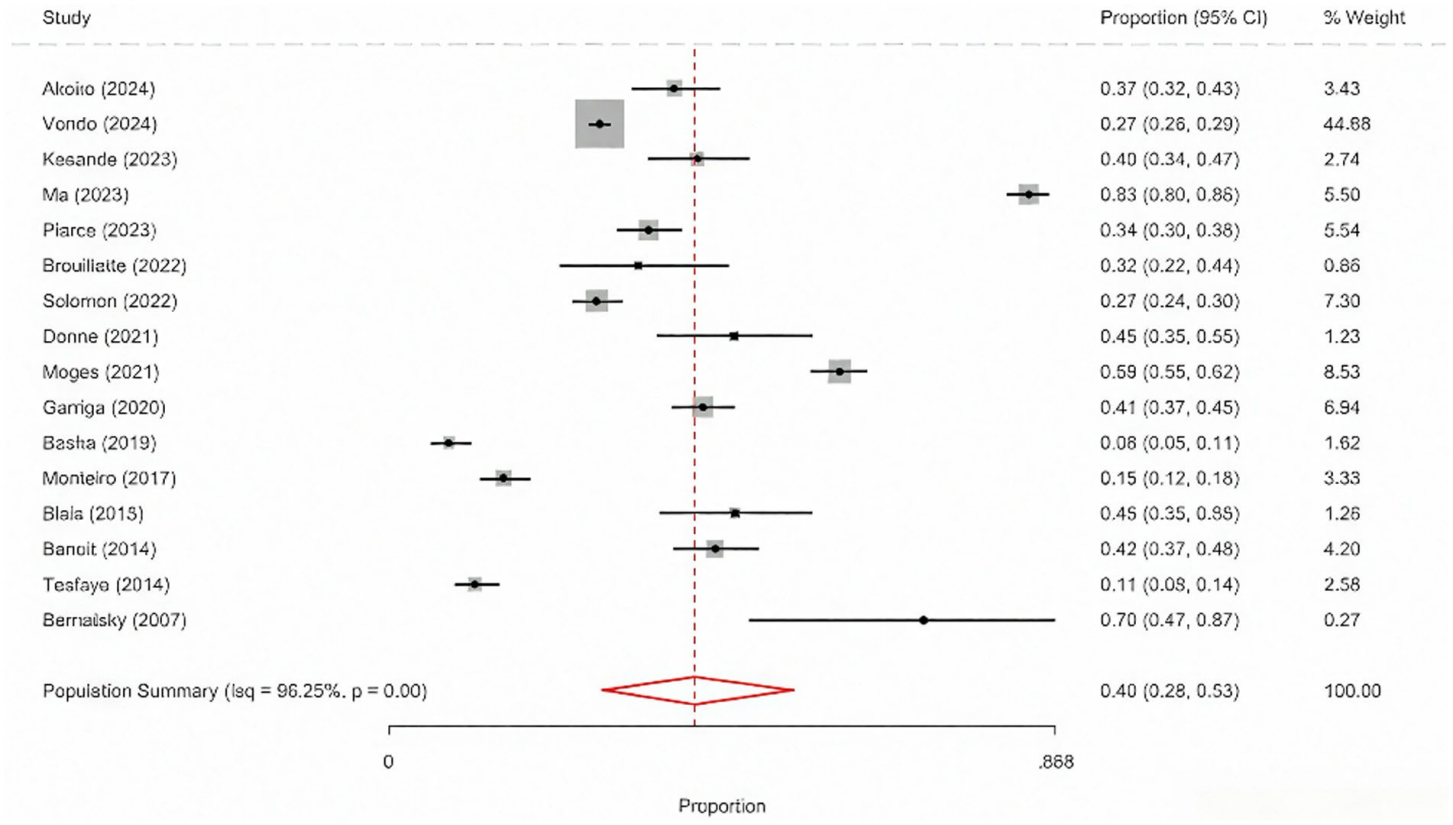
Forest plot of the pooled prevalence of PD in PLWH using GLMM.

The pooled prevalence of PD was 0.40 (95% CI: 0.28–0.53; Figure 2), with substantial heterogeneity (*τ^2^* = 0.94; *I^2^* = 96.25%, *p* < 0.001; individual study estimates: 0.08–0.83). Sensitivity analyses confirmed the robustness of this estimate: a HKSJ random-effects analysis yielded a back-transformed prevalence of 0.36 (95% CI: 0.26–0.48), and leave-one-out analyses produced estimates ranging from 36.0 to 41.0%, all consistent with the primary result (see Supplementary material S5).

#### 3.6.2 Subgroup analyses

Subgroup analyses were conducted by study period, geographic region, PD tool category, and population type (Table 2). Prevalence estimates varied across subgroups: from 0.31 (2015–2019) to 0.53 (post-2019) by era; from 0.35 (Europe/North America) to 0.83 (Asia) by region; from 0.29 (symptom tools) to 0.83 (HIV-specific tool) by PD tool; and from 0.38 (general PLWH) to 0.42 (special subpopulations) by population type. Notably, the Asia subgroup and the HIV-specific PD tool subgroup were each represented by a single study. In addition, we further conducted supplementary subgroup analyses by COVID-19 period and economic region, with forest plots presented in Supplementary material S6.

**Table 2:** Subgroup analysis of the prevalence of PD in PLWH.

Characteristics	Study (*n*)	PD (*n*)	PLWH (*n*)	Pooled prevalence (%)	95%CI (%)	Heterogeneity	
						*I^2^* (%)	*p*-value
Era							
Pre 2015	7	761	2,296	40.0	25–56	91.81	<0.001
2015 to 2019	6	2005	6,968	31.0	15–51	98.36	<0.001
Post 2019	3	706	940	53.0	24–82	95.77	<0.001
Region							
Sub-saharan Africa	8	2076	7,032	37.0	20–55	95.50	<0.001
Europe and North America	7	759	2,407	35.0	25–48	90.18	<0.001
Asia	1	637	765	83.0	80–86	NA	NA
PD tool							
General distress tools	9	2,393	7,432	40.0	27–56	94.81	<0.001
HIV specific PD tool	1	637	765	83.0	80–86	NA	NA
Symptom tools	6	442	2007	29.0	17–47	92.63	<0.001
Population							
General PLWH	9	2,502	7,599	38.0	22–57	97.81	<0.001
Special subpopulation	7	970	2,605	42.0	27–59	92.13	<0.001
COVID-19							
No	13	2,766	9,264	36.0	24–49	95.46	<0.001
Yes	3	706	940	53.0	24–82	95.77	<0.001
Economic regions							
LIC	4	583	1835	31.0	10–59	98.35	<0.001
LMIC	3	287	796	42.0	27–66	76.91	<0.001
UMIC	2	1843	5,166	36.0	34–37	NA	NA
HIC	7	759	2,407	35.0	25–48	90.18	<0.001

I^2^ was not calculated for subgroups with ≤2 studies due to instability, marked as NA.

LIC, Low-income country; LMIC, Lower-middle-income country; UMIC, Upper-middle-income country; HIC, High-income country.

#### 3.6.3 Meta-regression

Univariable meta-regression was performed to examine whether selected study-level characteristics were associated with between-study heterogeneity in PD prevalence (Table 3). Geographic region (*p* = 0.0312) and PD tool category (*p* = 0.0138) were associated with prevalence variation, while era, population type, COVID-19 period, and economic region were not. These findings suggest that geographic context and measurement approach may explain part of the observed heterogeneity. However, they should be interpreted cautiously because the number of included studies was small, some categories were represented by only one study, and substantial residual heterogeneity remained.

**Table 3:** Univariable meta-regression of the prevalence of PD in PLWH.

Characteristics	*β*	SE	*p-*value	95% CI	Adjusted *R*^2^
Era			0.166		10.27%
Post 2019	1.274	0.677	0.06	−0.053 to 2.602	
Pre 2015	0.523	0.532	0.326	−0.520 to 1.565	
Regions			0.0312		26.69%
Europe and North America	−2.257	0.918	0.014	−4.057 to −0.458	
Sub-Saharan Africa	−2.38	0.911	0.009	−4.166 to −0.594	
PD tool			0.0138		32.29%
HIV Specific PD Tool	2.136	0.871	0.014	0.429 to 3.842	
Symptom Tools	−0.47	0.441	0.287	−1.334 to 0.395	
Population			0.5258		0.00%
Special subpopulation	0.331	0.521	0.526	−0.691 to 1.352	
COVID-19 period			0.1031		11.40%
Yes	0.997	0.612	0.103	−0.202 to 2.196	
Economic region			0.3396		1.49%
LIC	−0.485	0.626	0.438	−1.712 to 0.741	
LMIC	0.473	0.701	0.500	−0.901 to 1.847	
UMIC	0.962	0.378	0.227	−0.598 to 2.522	

Reference categories were 2015–2019 for era, Asia for region, general distress tools for PD tool category, general PLWH for population type, non-COVID-19 period for COVID-19 period, and HIC for economic region.

#### 3.6.4 Publication Bias

**Figure 3:**
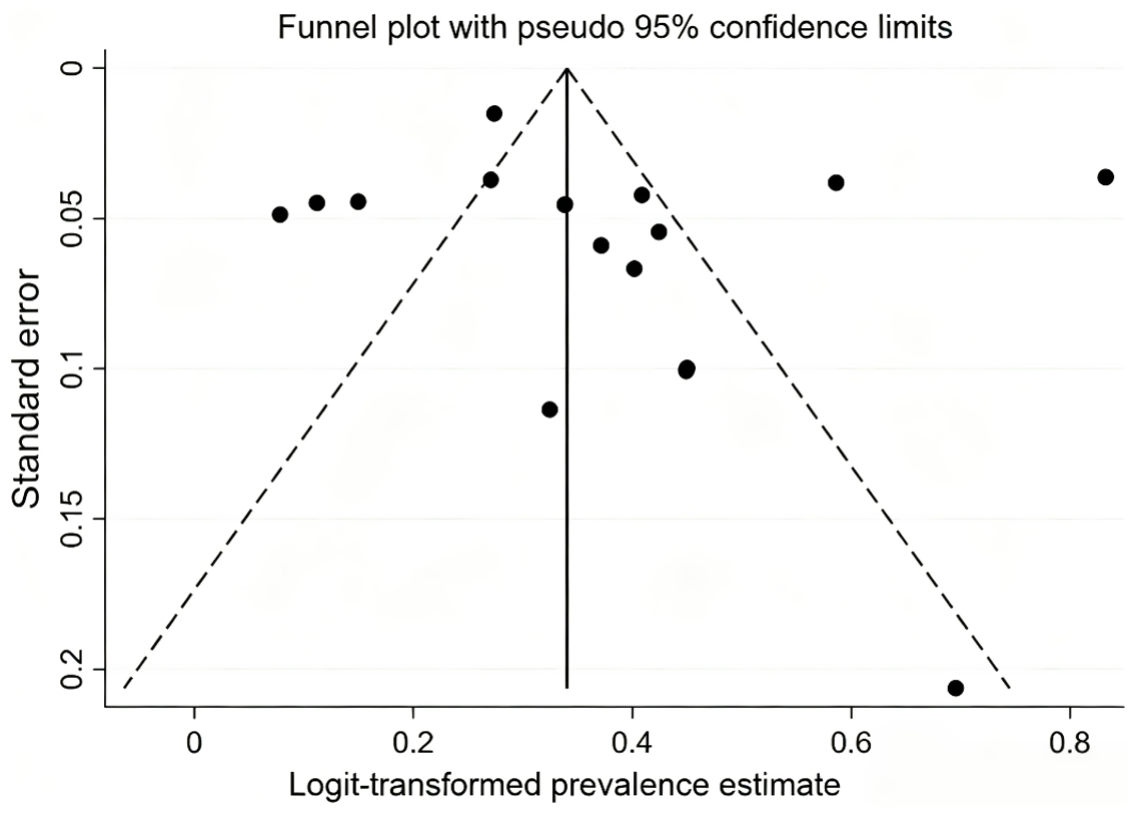
Funnel plot assessing publication bias for the overall pooled prevalence of PD among PLWH. Each dot represents one included study. The horizontal axis shows the logit-transformed prevalence estimate, and the vertical axis shows its standard error.

Egger’s test (*t* = 0.72, *p* = 0.484) and Begg’s test (*z* = 0.54, *p* = 0.589) indicated a low probability of publication bias for the overall prevalence. Publication bias was not assessed for individual associated factors due to the limited number of studies per factor. A funnel plot for the overall prevalence is provided in Figure 3.

#### 3.6.5 Factors associated with PD

To align the synthesis of associated factors with the BPS framework, study-reported correlates were organized into biological, psychological/behavioral, and social or contextual domains. Within the biological domain, low CD4 count was associated with higher odds of PD (OR = 2.59, 95% CI: 1.62–4.13). Within the psychological/behavioral domain, poor ART adherence was associated with higher odds of PD (OR = 4.55, 95% CI: 1.96–10.54), whereas self-perceived health showed a borderline association (OR = 2.13, 95% CI: 1.00–4.54). Within the social or contextual domain, non-disclosure of HIV status (OR = 4.95, 95% CI: 3.23–7.58) and female sex (OR = 2.26, 95% CI: 1.67–3.07) were associated with higher odds of PD, whereas being married was associated with lower odds of PD (OR = 0.35, 95% CI: 0.26–0.47). Age, current job, and education level were not significantly associated with PD in the pooled analyses (Table 4). Because the definitions and reference categories of several candidate correlates varied across original studies, these pooled estimates should be interpreted as approximate associations rather than fully standardized effects. Forest plots for associated factors are provided in Supplementary material S7.

**Table 4:** Pooled associations between candidate correlates and PD (ORs, 95% CIs).

Influencing factors	Study (*n*)	Meta-analysis			Heterogeneity	
		OR	95% CI	*p* value	*I^2^* (%)	*p*
Age	6	1.18	0.82–1.69	0.369	79.4	<0.001
Female sex	5	2.26	1.67–3.07	**<0.001**	30.4	0.219
Marital status	4	0.35	0.26–0.47	**<0.001**	9.5	0.345
Current job	6	1.00	0.33–3.01	0.996	86.2	<0.001
ART adherence	4	4.55	1.96–10.54	**<0.001**	88.4	<0.001
CD4 count (cells/mm^3^)	3	2.59	1.62–4.13	**<0.001**	0.0	0.615
Education level	4	0.75	0.37–1.54	0.435	81.0	<0.001
Disclosure	4	4.95	3.23–7.58	**<0.001**	0.0	0.672
Self-perceived health	2	2.13	1.00–4.54	0.05	84.5	0.002

ORs were pooled according to the contrast definitions reported in the original studies. Age was synthesized using the study-reported age contrast or age-unit definition. For female sex, the comparison was female versus male, and disclosure generally reflected non-disclosure versus disclosure of HIV status. For marital status, CD4 count, education, employment, ART adherence, and self-perceived health, reference groups, cutoff values, or operational definitions varied across studies. Therefore, pooled ORs should be interpreted as approximate cross-study associations rather than fully standardized effects. Bold values indicate statistical significance (*p* < 0.05).

## 4 Discussion

Guided by the integrated BPS-TMSC framework, this updated systematic review and meta-analysis synthesized global evidence on the prevalence, measurement variation, and associated factors of PD among PLWH. The pooled prevalence was 40.0%, with a broadly similar estimate in sensitivity analysis, suggesting that PD remains a substantial mental health burden in HIV care. However, heterogeneity was considerable, and the certainty of evidence was low to very low. PD tool category and geographic region were the study-level characteristics most clearly associated with prevalence variation, whereas era, population type, COVID-19 period, and economic region were not consistently associated with between-study differences. The pooled associations involving poor ART adherence, non-disclosure, low CD4 count, female sex, and marital status may help identify groups requiring greater psychosocial attention, but they should be interpreted as non-causal associations because most included studies were cross-sectional and the evidence certainty was limited.

The theory-informed approach provides an additional contribution beyond estimating pooled prevalence. Compared with previous reviews that mainly summarized prevalence, the integrated BPS-TMSC framework helped organize associated factors into biological, psychological/behavioral, and social domains and interpret how HIV-related stressors may interact with coping resources. In this review, the framework was not used to test causal pathways but to provide an interpretive structure for understanding why factors such as CD4 count, ART adherence, disclosure, marital status, and female sex may cluster around PD risk.

The pooled prevalence of 40.0% suggests that PD is a substantial component of the lived experience of PLWH, not merely a frequent comorbidity. This estimate is slightly lower than reported in the previous meta-analysis (43.7%) (18), which may reflect differences in eligibility criteria, study composition, measurement approach, and statistical methods. The overlap with the previous review was limited because the present review additionally required extractable associated-factor information under the current operational definition of PD. To examine whether newer studies drove this difference, we conducted an additional sensitivity analysis restricted to the seven studies published in 2022 or later; the pooled prevalence was 0.41 (95% CI: 0.26–0.59), close to the overall estimate. Thus, the slightly lower prevalence observed in the present review is unlikely to be explained solely by the inclusion of newer studies. Over half of the included studies were conducted in recent years, a period of improved public awareness (39) and, in some settings, reduced HIV-related stigma (40). Nevertheless, the observed burden remains clearly higher than that in the general population (13.3–17.9%) (41, 42) and comparable to estimates in cancer survivors (32–50%) (43–45) and individuals recovering from COVID-19 (36%) (46). These comparisons support the continuing relevance of PD as a mental health concern in chronic and life-altering conditions, including HIV. However, the marked heterogeneity indicates that this burden is not uniform across settings but is highly sensitive to study context and measurement approach.

Variation in PD tool category appears to be an important contributor to the observed heterogeneity. Subgroup and meta-regression analyses showed that prevalence estimates differed across general distress tools, symptom tools, and the HIV-specific instrument. In subgroup analysis, symptom tools showed a lower pooled prevalence, whereas the single study using an HIV-specific tool showed the highest estimate. This pattern likely reflects differences in the underlying constructs captured by the instruments. Symptom-oriented tools, such as HADS (12, 37) or PHQ-4 (36), primarily capture anxiety, depressive, or related symptom manifestations and may therefore identify a narrower subset of distress. General distress tools capture broader emotional suffering, while the HIV-specific HRPDS was designed to capture HIV-related distress dimensions, including illness-coping, identity, disclosure, and social interaction distress (9, 32). These findings suggest that generic or symptom-oriented tools may underestimate HIV-specific distress when identity and disclosure-related suffering is central to patients’ experiences. However, this should not be interpreted as evidence of instrument superiority, because the HIV-specific tool category was represented by only one study. Rather, different tools appear to capture partially overlapping but non-equivalent dimensions of PD.

Geographic region was also associated with prevalence variation, although this pattern requires cautious interpretation. In subgroup analysis, the Asia subgroup showed the highest prevalence estimate, whereas Sub-Saharan Africa and Europe/North America showed broadly similar pooled estimates. However, the higher estimate for Asia was based on a single study conducted in China (32), which also used the HIV-specific HRPDS instrument. Therefore, the apparent regional difference may partly reflect measurement approach rather than geographic context alone. This overlap between region and tool category limits the extent to which the higher estimate can be interpreted as a stable regional pattern. For Sub-Saharan Africa and Europe/North America, the pooled estimates were relatively similar, suggesting that regional differences may not operate in a simple high-versus-low pattern. Instead, geographic region is likely to act as a proxy for broader contextual conditions, including health system capacity, integration of mental health and HIV services, psychosocial support infrastructure, stigma environment, and local measurement or reporting practices (7, 19). These contextual factors are unlikely to operate independently and may interact with measurement approach, population composition, and care setting. Thus, while regional context may contribute to prevalence variation, the present evidence does not support a stable regional hierarchy of PD burden.

The BPS-based organization of associated factors in the preceding text suggests that PD among PLWH is linked to biological vulnerability, psychological/behavioral adaptation, and social or contextual conditions. The TMSC framework further helps interpret these associations as reflecting a possible imbalance between HIV-related stressors, appraisal processes, and available coping resources. Guided by this integrated interpretation, we propose a hypothetical risk-resilience model to understand the observed associations. Non-disclosure of HIV status (OR = 4.95) may be linked to isolation from limited social support and persistent hypervigilance due to fear of stigma and accidental disclosure (32, 34). This co-occurs with the protective effect of being married (OR = 0.35), which may operate through stable emotional support (47), dyadic coping (48), and shared economic resources (49), though this benefit may be attenuated by traditional gender roles (33). The elevated PD risk among female PLWH (OR = 2.26) may stem from biosocial vulnerabilities, including hormonal fluctuations (50), pregnancy-related stressors (51), moral scrutiny (52), and intimate partner violence (53). Furthermore, the strong associations of poor ART adherence (OR = 4.55) and low CD4 count (OR = 2.59) with PD are consistent with a hypothesized “psycho-adherence” feedback loop (54), in which distress may be associated with lower medication motivation (55) and immunological decline (56), potentially forming a bidirectional pattern that exacerbates distress through biological (e.g., neuroinflammation) and psychological (e.g., disease anxiety) pathways. This process may be modulated by inadequate social support, illustrating dynamic biopsychosocial interplay. Collectively, these findings are compatible with a framework in which PD emerges when appraised HIV-related stressors (e.g., stigma, immune decline) exceed coping resources (e.g., marital support, safe disclosure environments), potentially alongside maladaptive behaviors (e.g., poor ART adherence). It is critical to emphasize that these interpretations, grounded in theoretical frameworks, derive from observational data and do not permit causal inferences—a caution further warranted by the GRADE assessment rating the certainty of evidence as low or very low.

These findings also suggest directions for future measurement development. Existing tools only partially capture the multidimensional distress process implied by the BPS-TMSC framework. Future studies should consider developing and validating PLWH-specific distress instruments that assess not only affective symptoms but also HIV-related identity distress, disclosure concerns, stigma-related vigilance, illness-coping burden, treatment engagement, and social interaction difficulties. Such instruments should be tested across cultural and service settings, with attention to measurement invariance, clinically meaningful cutoffs, and feasibility for routine HIV care.

### 4.1 Implications

The present findings have several practical implications, although they should be interpreted cautiously given the substantial heterogeneity and the low to very low certainty of evidence. At the clinical follow-up level, HIV services may benefit from incorporating a brief first-line check for PD, particularly for patients with poor ART adherence, low CD4 count, non-disclosure of HIV status, or other indicators of psychosocial vulnerability. This is consistent with the broader direction of integrating mental health care into non-specialized health services, as emphasized in the WHO mhGAP guidance (19). Rather than requiring a full psychiatric assessment at every visit, a brief validated screening step may help identify individuals who warrant further psychosocial review. At the psychosocial support level, PD management is likely to be most useful when linked to concrete follow-up actions rather than treated as a stand-alone screening exercise. Patients screening positive may benefit from follow-up focused on adherence counseling, disclosure-related support, family or partner communication, and referral to psychological or social work services where available (47–49, 52–55). This may be particularly relevant for women, newly diagnosed individuals, and others facing layered psychosocial pressures. At the service and policy level, implementation should remain context-sensitive rather than rely on a single universal model. In resource-constrained settings, a realistic priority may be to strengthen case recognition and referral within existing HIV services. In better-resourced settings, the priority may be to improve coordination between HIV care, psychological support, and community-based services so that PD screening is linked to feasible follow-up options. Accordingly, psychosocial care for PLWH is likely to be more feasible and useful when matched to local service capacity and patient needs.

### 4.2 Limitations

Several limitations should be acknowledged. First, between-study heterogeneity remained substantial throughout the analyses and was only partially explained by the available study-level characteristics. To mitigate this issue, we conducted pre-specified subgroup analyses, univariable meta-regression, and sensitivity analyses, and interpreted pooled estimates cautiously rather than as uniform global values. Second, the number of included studies was relatively small, and some subgroup categories, particularly the Asia subgroup and the HIV-specific PD tool category, were represented by only a single study. We therefore avoided making stable regional or instrument-level hierarchy claims. Third, PD was measured using instruments with different construct coverage and cutoff thresholds. To address this issue, we applied a hierarchical operational definition, classified tools into HIV-specific, general distress, and symptom-oriented categories, and examined measurement approach as a potential source of heterogeneity. Fourth, several candidate correlates were defined inconsistently across original studies, limiting the standardization of pooled ORs. We therefore interpreted these estimates as approximate cross-study associations rather than fully standardized effects. Fifth, most included studies were cross-sectional, which precludes causal inference. We attempted to mitigate this limitation by consistently using non-causal language and by positioning the BPS-TMSC framework as an interpretive structure rather than a causal testing model. Finally, the overall certainty of evidence was low to very low; accordingly, the current evidence is better understood as informing hypothesis generation, risk identification, measurement refinement, and context-sensitive care priorities rather than definitive cross-context conclusions.

## 5 Conclusion

This updated systematic review and meta-analysis suggests that psychological distress remains common among PLWH, with a pooled prevalence of approximately 40% in the primary analysis and a broadly similar estimate in sensitivity analysis. Prevalence estimates varied across study contexts and measurement approaches, with PD tool category and geographic region identified as study-level characteristics associated with prevalence variation. However, substantial residual heterogeneity remained, and some subgroup findings were based on sparse categories or single studies. Pooled analyses of associated factors suggested that poor ART adherence, non-disclosure, low CD4 count, and female sex were associated with higher odds of PD, whereas being married was associated with lower odds. These associations should not be interpreted as establishing causal direction, given that most included studies were cross-sectional. Overall, the present findings support the view that PD is an important and globally relevant burden among PLWH, but they should be interpreted cautiously due to considerable heterogeneity and low to very low certainty of evidence. Rather than providing definitive cross-context conclusions, this review offers an updated evidence base for identifying vulnerable contexts, refining measurement strategies, and guiding focused research on the biopsychosocial burden of distress in HIV care.

## Data Availability

The original contributions presented in the study are included in the article/Supplementary material, further inquiries can be directed to the corresponding author.
