# Supplementary material for: Global prevalence and biopsychosocial correlates of psychological distress among people living with HIV: an updated theory-informed meta-analysis
Source: Front Public Health. 2026 Jun 19;14:1832557. doi: 10.3389/fpubh.2026.1832557 (PMC13328019; doi:10.3389/fpubh.2026.1832557)

***Supplementary Material***

Supplementary 1 PRISMA 2020 Checklist

| **Section and Topic** | **Item #** | **Checklist item** | **Location where item is reported** |
| --- | --- | --- | --- |
| **TITLE** | | |  |
| Title | 1 | Identify the report as a systematic review. | Title |
| **ABSTRACT** | | |  |
| Abstract | 2 | See the PRISMA 2020 for Abstracts checklist. | Abstract |
| **INTRODUCTION** | | |  |
| Rationale | 3 | Describe the rationale for the review in the context of existing knowledge. | Introduction |
| Objectives | 4 | Provide an explicit statement of the objective(s) or question(s) the review addresses. | Introduction |
| **METHODS** | | |  |
| Eligibility criteria | 5 | Specify the inclusion and exclusion criteria for the review and how studies were grouped for the syntheses. | Methods 2.4 |
| Information sources | 6 | Specify all databases, registers, websites, organisations, reference lists and other sources searched or consulted to identify studies. Specify the date when each source was last searched or consulted. | Methods 2.2 |
| Search strategy | 7 | Present the full search strategies for all databases, registers and websites, including any filters and limits used. | Methods 2.2 |
| Selection process | 8 | Specify the methods used to decide whether a study met the inclusion criteria of the review, including how many reviewers screened each record and each report retrieved, whether they worked independently, and if applicable, details of automation tools used in the process. | Methods 2.5 |
| Data collection process | 9 | Specify the methods used to collect data from reports, including how many reviewers collected data from each report, whether they worked independently, any processes for obtaining or confirming data from study investigators, and if applicable, details of automation tools used in the process. | Methods 2.5 |
| Data items | 10a | List and define all outcomes for which data were sought. Specify whether all results that were compatible with each outcome domain in each study were sought (e.g. for all measures, time points, analyses), and if not, the methods used to decide which results to collect. | Methods 2.5 |
|  | 10b | List and define all other variables for which data were sought (e.g. participant and intervention characteristics, funding sources). Describe any assumptions made about any missing or unclear information. | Methods 2.5&2.8 |
| Study risk of bias assessment | 11 | Specify the methods used to assess risk of bias in the included studies, including details of the tool(s) used, how many reviewers assessed each study and whether they worked independently, and if applicable, details of automation tools used in the process. | Methods 2.6 |
| Effect measures | 12 | Specify for each outcome the effect measure(s) (e.g. risk ratio, mean difference) used in the synthesis or presentation of results. | Methods 2.8 |
| Synthesis methods | 13a | Describe the processes used to decide which studies were eligible for each synthesis (e.g. tabulating the study intervention characteristics and comparing against the planned groups for each synthesis (item #5)). | Methods 2.8 |
|  | 13b | Describe any methods required to prepare the data for presentation or synthesis, such as handling of missing summary statistics, or data conversions. | Methods 2.8 |
|  | 13c | Describe any methods used to tabulate or visually display results of individual studies and syntheses. | Methods 2.8 |
|  | 13d | Describe any methods used to synthesize results and provide a rationale for the choice(s). If meta-analysis was performed, describe the model(s), method(s) to identify the presence and extent of statistical heterogeneity, and software package(s) used. | Methods 2.8 |
|  | 13e | Describe any methods used to explore possible causes of heterogeneity among study results (e.g. subgroup analysis, meta-regression). | Methods 2.8 |
|  | 13f | Describe any sensitivity analyses conducted to assess robustness of the synthesized results. | Methods 2.8 |
| Reporting bias assessment | 14 | Describe any methods used to assess risk of bias due to missing results in a synthesis (arising from reporting biases). | Methods 2.8 |
| Certainty assessment | 15 | Describe any methods used to assess certainty (or confidence) in the body of evidence for an outcome. | Methods 2.7 |
| **RESULTS** | | |  |
| Study selection | 16a | Describe the results of the search and selection process, from the number of records identified in the search to the number of studies included in the review, ideally using a flow diagram. | Results 3.1 |
|  | 16b | Cite studies that might appear to meet the inclusion criteria, but which were excluded, and explain why they were excluded. | Results 3.1 |
| Study characteristics | 17 | Cite each included study and present its characteristics. | Results 3.2 |
| Risk of bias in studies | 18 | Present assessments of risk of bias for each included study. | Results 3.4 |
| Results of individual studies | 19 | For all outcomes, present, for each study: (a) summary statistics for each group (where appropriate) and (b) an effect estimate and its precision (e.g. confidence/credible interval), ideally using structured tables or plots. | Results 3.6 |
| Results of syntheses | 20a | For each synthesis, briefly summarise the characteristics and risk of bias among contributing studies. | Results 3.2&3.4 |
|  | 20b | Present results of all statistical syntheses conducted. If meta-analysis was done, present for each the summary estimate and its precision (e.g. confidence/credible interval) and measures of statistical heterogeneity. If comparing groups, describe the direction of the effect. | Results 3.6 |
|  | 20c | Present results of all investigations of possible causes of heterogeneity among study results. | Results 3.6 |
|  | 20d | Present results of all sensitivity analyses conducted to assess the robustness of the synthesized results. | Results 3.6.1 |
| Reporting biases | 21 | Present assessments of risk of bias due to missing results (arising from reporting biases) for each synthesis assessed. | Results 3.6.4 |
| Certainty of evidence | 22 | Present assessments of certainty (or confidence) in the body of evidence for each outcome assessed. | Results 3.5 |
| **DISCUSSION** | | |  |
| Discussion | 23a | Provide a general interpretation of the results in the context of other evidence. | Discussion P1 |
|  | 23b | Discuss any limitations of the evidence included in the review. | Discussion 4.2 |
|  | 23c | Discuss any limitations of the review processes used. | Discussion 4.2 |
|  | 23d | Discuss implications of the results for practice, policy, and future research. | Discussion 4.1 |
| **OTHER INFORMATION** | | |  |
| Registration and protocol | 24a | Provide registration information for the review, including register name and registration number, or state that the review was not registered. | Methods 2.1 |
|  | 24b | Indicate where the review protocol can be accessed, or state that a protocol was not prepared. | Methods 2.1 |
|  | 24c | Describe and explain any amendments to information provided at registration or in the protocol. | - |
| Support | 25 | Describe sources of financial or non-financial support for the review, and the role of the funders or sponsors in the review. | Funding |
| Competing interests | 26 | Declare any competing interests of review authors. | Conflict of interest |
| Availability of data, code and other materials | 27 | Report which of the following are publicly available and where they can be found: template data collection forms; data extracted from included studies; data used for all analyses; analytic code; any other materials used in the review. | Data availability statement |

Supplementary 2 Search strategies

2.1 Search strategy in PubMed/Medline

**Search time: 2025-06-20 20:00**

| Search | Query | Items found |
| --- | --- | --- |
| #1 | ("Acquired Immunodeficiency Syndrome"[MeSH Terms] OR "HIV"[MeSH Terms] OR "HIV Infections"[MeSH Terms]) | 366,216 |
| #2 | "acquired immunodeficiency syndrome"[Title/Abstract] OR "AIDS"[Title/Abstract] OR "acquired immune deficiency syndrome"[Title/Abstract] OR "HIV"[Title/Abstract] OR "hiv infections"[Title/Abstract] OR "human immunodeficiency virus"[Title/Abstract] OR "aids virus"[Title/Abstract] OR "acquired immunodeficiency syndrome virus"[Title/Abstract] OR "people living with hiv aids"[Title/Abstract] OR "persons living with hiv"[Title/Abstract] | 495,902 |
| #3 | #1 OR #2 | 534,239 |
| #4 | "Psychological Distress"[MeSH Terms] | 9,028 |
| #5 | "psychological distress"[Title/Abstract] OR "emotional distress"[Title/Abstract] OR "hiv related psychological distress"[Title/Abstract] OR "Distress"[Title/Abstract] | 176,675 |
| #6 | #4 OR #5 | 180,061 |
| #7 | "Risk Factors"[MeSH Terms] OR "Protective Factors"[MeSH Terms] | 1,045,918 |
| #8 | "risk factors"[Title/Abstract] OR "protective factors"[Title/Abstract] OR "influencing factors"[Title/Abstract] OR "impact factors"[Title/Abstract] OR "Relation"[Title/Abstract] OR "Correlate"[Title/Abstract] OR "Predictor"[Title/Abstract] | 1,705,311 |
| #9 | #7 OR #8 | 2,361,876 |
| #10 | #3 AND #6 AND #9 | 482 |

2.2 Search strategy in EMBASE (OVID)

**Search time: 2025-06-20 20:30**

| Search | Query | Items found |
| --- | --- | --- |
| #1 | ('acquired immune deficiency syndrome'/exp OR 'acquired immune deficiency syndrome')AND [embase]/lim | 374,054 |
| #2 | ('acquired human immunodeficiency syndrome':ab,ti OR 'acquired immune deficiency disease syndrome':ab,ti OR 'acquired immuno-deficiency syndrome':ab,ti OR 'acquired immunodeficiency disease syndrome':ab,ti OR 'acquired immunodeficiency syndrome':ab,ti OR 'acquired immunodeficiency virus syndrome':ab,ti OR 'aids':ab,ti OR 'aquired immune deficiency syndrome':ab,ti OR 'aquired immunodeficiency syndrome':ab,ti OR 'hiv/aids':ab,ti OR 'human immune deficiency virus/acquired immune deficiency syndrome':ab,ti OR 'human immunodeficiency virus infection/acquired immunodeficiency syndrome':ab,ti OR 'immunodeficiency, acquired':ab,ti OR 'acquired immune deficiency syndrome':ab,ti)AND [embase]/lim | 144,703 |
| #3 | ('human immunodeficiency virus'/exp OR 'human immunodeficiency virus')AND [embase]/lim | 450,663 |
| #4 | ('aids associated lentivirus':ab,ti OR 'aids associated retrovirus':ab,ti OR 'aids associated virus':ab,ti OR 'aids related virus':ab,ti OR 'aids virus':ab,ti OR 'hiv':ab,ti OR 'human immuno deficiency virus':ab,ti OR 'immunodeficiency associated virus':ab,ti OR 'lav':ab,ti OR lav:ab,ti) AND aids:ab,ti OR 'lymphadenopathy associated retrovirus':ab,ti OR 'lymphadenopathy associated virus':ab,ti OR 'virus, lymphadenopathy associated':ab,ti OR 'human immunodeficiency virus':ab,ti)AND [embase]/lim | 151,798 |
| #5 | ('aids patient'/exp OR 'aids patient')AND [embase]/lim | 9,328 |
| #6 | ('acquired immune deficiency syndrome patient':ab,ti OR 'aids sufferer':ab,ti OR 'aids victim':ab,ti OR 'hiv/aids sufferer':ab,ti OR 'hiv/aids victim':ab,ti OR 'patient living with hiv/aids':ab,ti OR 'patient with aids':ab,ti OR 'patient with hiv/aids':ab,ti OR 'patients living with hiv/aids':ab,ti OR 'patients with aids':ab,ti OR 'people living with hiv aids':ab,ti OR 'people living with hiv/aids':ab,ti OR 'person living with hiv/aids':ab,ti OR 'persons living with hiv/aids':ab,ti OR plwha:ab,ti)AND [embase]/lim | 12,415 |
| #7 | #1 OR #2 OR #3 OR #4 OR #5 OR #6 | 766,052 |
| #8 | ('emotional stress'/exp OR 'emotional stress')AND [embase]/lim | 35,502 |
| #9 | ('emotional burden':ab,ti OR 'emotional distress':ab,ti OR 'emotional overload':ab,ti OR 'emotional pressure':ab,ti OR 'emotional shock':ab,ti OR 'emotional tension':ab,ti OR 'stress, emotional':ab,ti OR 'emotional stress':ab,ti)AND [embase]/lim | 17,938 |
| #10 | ('psychological distress':ab,ti OR 'emotional distress':ab,ti OR distress:ab,ti OR 'hiv-related psychological distress':ab,ti)AND [embase]/lim | 186,945 |
| #11 | #8 OR #9 OR #10 | 212,011 |
| #12 | ('risk factor'/exp OR 'risk factor')AND [embase]/lim | 1,450,976 |
| #13 | ('relative risk':ab,ti OR 'risk factors':ab,ti OR 'risk factor':ab,ti)AND [embase]/lim | 1,177,579 |
| #14 | ('risk factors':ab,ti OR 'protective factors':ab,ti OR 'influencing factors':ab,ti OR 'impact factors':ab,ti OR relation:ab,ti OR correlate:ab,ti OR predictor:ab,ti)AND [embase]/lim | 1,870,474 |
| #15 | #12 OR #13 OR #14 | 2,678,534 |
| #16 | #7 AND #11 AND #15 | 808 |

2.3 Search strategy in Web of Science - Web of Science Core Collection

**Search time: 2025-06-20 21:00**

| Search | Query | Items found |
| --- | --- | --- |
| #1 | TS=(Acquired Immunodeficiency Syndrome or AIDS or Acquired Immune Deficiency Syndrome or HIV or HIV Infections or Human Immunodeficiency Virus or AIDS Virus or Acquired Immunodeficiency Syndrome Virus or People living with HIV/AIDS or Persons living with HIV) | 640,149 |
| #2 | TS=(Psychological Distress or Emotional Distress or Distress or HIV-related Psychological Distress) | 163,584 |
| #3 | TS=(Risk factors or Protective factors or Influencing factors or Impact factors or Relation or Correlate or Predictor) | 4,333,554 |
| #4 | #1 AND #2 AND #3 | 1,405 |

2.4 Search strategy in CINHAL (EBSCO)

**Search time: 2025-06-20 21:30**

| Search | Query | Items found |
| --- | --- | --- |
| S1 | MH "Human Immunodeficiency Virus+" | 11,375 |
| S2 | MH "Acquired Immunodeficiency Syndrome" | 18,001 |
| S3 | MH "AIDS Patients" | 2,964 |
| S4 | XB (Acquired Immunodeficiency Syndrome or AIDS or Acquired Immune Deficiency Syndrome or HIV or HIV Infections or Human Immunodeficiency Virus or AIDS Virus or Acquired Immunodeficiency Syndrome Virus or People living with HIV/AIDS or Persons living with HIV) | 140,523 |
| S5 | S1 OR S2 OR S3 OR S4 | 147,209 |
| S6 | MH "Psychological Distress" | 10,074 |
| S7 | XB (Psychological Distress or Emotional Distress or Distress or HIV-related Psychological Distress) | 70,388 |
| S8 | S6 OR S7 | 73,227 |
| S9 | MH "Risk Factors+" | 229,557 |
| S10 | MH "Risk Factors+" | 56,656 |
| S11 | XB (Risk factors or Protective factors or Influencing factors or Impact factors or Relation or Correlate or Predictor) | 608,669 |
| S12 | S9 OR S10 OR S11 | 792,642 |
| S13 | S5 AND S8 AND S12 | 389 |

2.5 Search strategy in Cochrane Library

**Search time: 2025-06-20 22:00**

| Search | Query | Items found |
| --- | --- | --- |
| #1 | MeSH descriptor: [Acquired Immunodeficiency Syndrome] explode all trees | 2,492 |
| #2 | MeSH descriptor: [HIV] explode all trees | 4,146 |
| #3 | MeSH descriptor: [HIV Infections] explode all trees | 17,956 |
| #4 | (Acquired Immunodeficiency Syndrome):ti,ab,kw OR (AIDS):ti,ab,kw OR (Acquired Immune Deficiency Syndrome):ti,ab,kw OR (HIV):ti,ab,kw OR (HIV Infections):ti,ab,kw | 40,113 |
| #5 | (Human Immunodeficiency Virus):ti,ab,kw OR (AIDS Virus):ti,ab,kw OR (Acquired Immunodeficiency Syndrome Virus):ti,ab,kw OR (People living with HIV):ti,ab,kw OR (Persons living with HIV):ti,ab,kw | 15,809 |
| #6 | #1 OR #2 OR #3 OR #4 OR #5 | 40,758 |
| #7 | MeSH descriptor: [Psychological Distress] explode all trees | 630 |
| #8 | (Emotional Distress):ti,ab,kw OR (HIV-related Psychological Distress):ti,ab,kw OR (Distress):ti,ab,kw OR (Psychological Distress):ti,ab,kw | 31,591 |
| #9 | #7 OR #8 | 31,749 |
| #10 | MeSH descriptor: [Risk Factors] explode all trees | 37,977 |
| #11 | MeSH descriptor: [Protective Factors] explode all trees | 276 |
| #12 | (Risk factors):ti,ab,kw OR (protective factors):ti,ab,kw OR (influencing factors):ti,ab,kw OR (impact factors):ti,ab,kw OR (Relation):ti,ab,kw | 150,671 |
| #13 | (Correlate):ti,ab,kw OR (Predictor):ti,ab,kw | 29,771 |
| #14 | #10 OR #11 OR #12 OR #13 | 174,738 |
| #15 | #6 AND #9 AND #14 | 90 |

2.6 Search strategy in PsycInfo

**Search time: 2025-06-20 22:30**

| Search | Query | Items found |
| --- | --- | --- |
| S1 | XB Acquired Immunodeficiency Syndrome or AIDS or Acquired Immune Deficiency Syndrome or HIV or HIV Infections or Human Immunodeficiency Virus or AIDS Virus or Acquired Immunodeficiency Syndrome Virus or People living with HIV/AIDS or Persons living with HIV | 79,998 |
| S2 | XB Psychological Distress or Emotional Distress or Distress or HIV-related Psychological Distress | 96,213 |
| S3 | XB Risk factors or Protective factors or Influencing factors or Impact factors or Relation or Correlate or Predictor | 774,600 |
| S4 | S1 AND S2 AND S3 | 647 |

2.7 Search strategy in CNKI

**Search time: 2025-06-20 23:00**

| Search | Query | Items found |
| --- | --- | --- |
| #1 | SU='Acquired Immunodeficiency Syndrome' / '获得性免疫缺陷综合征' OR SU='AIDS' / '艾滋病' OR SU='HIV' OR SU='Human Immunodeficiency Virus' / '人类免疫缺陷病毒' | 105,304 |
| #2 | SU='Psychological Distress' / '心理痛苦' OR SU='Emotional Distress' / '情绪困扰' | 2,795 |
| #3 | #1 AND #2 | 27 |

Supplementary 3 Quality assessment

3.1 AHRQ checklist

| **Item** | **Yes** | **No** | **Unclear** |
| --- | --- | --- | --- |
| 1. Define the source of information (survey, record review). |  |  |  |
| 2. List inclusion and exclusion criteria for exposed and unexposed subjects (cases and controls) or refer to previous publications. |  |  |  |
| 3. Indicate time period used for identifying patients. |  |  |  |
| 4. Indicate whether subjects were consecutive if not population-based. |  |  |  |
| 5. Indicate if evaluators of subjective components of study were masked to other aspects of the status of the participants. |  |  |  |
| 6. Describe any assessments undertaken for quality assurance purposes (e.g., test/retest of primary outcome measurements). |  |  |  |
| 7. Explain any patient exclusions from analysis. |  |  |  |
| 8. Describe how confounding was assessed and/or controlled. |  |  |  |
| 9. If applicable, explain how missing data were handled in the analysis. |  |  |  |
| 10. Summarize patient response rates and completeness of data collection. |  |  |  |
| 11. Clarify what follow-up, if any, was expected and the percentage of patients for which incomplete data or follow-up was obtained. |  |  |  |

3.2 The scores of all AHRQ checklist items in each cross-sectional study in this research

| **Study** | **Items and scores** | | | | | | | | | | | **Score** | **Level** |
| --- | --- | --- | --- | --- | --- | --- | --- | --- | --- | --- | --- | --- | --- |
|  | **1** | **2** | **3** | **4** | **5** | **6** | **7** | **8** | **9** | **10** | **11** |  |  |
| Vondo N (2024) | 1 | 1 | 1 | 0 | 1 | 0 | 1 | 1 | 0 | 1 | 0 | 7 | Moderate |
| Kesande C (2023) | 1 | 1 | 1 | 1 | 1 | 0 | 1 | 1 | 0 | 1 | 0 | 8 | High |
| Ma H Q (2023) | 1 | 1 | 1 | 0 | 1 | 0 | 1 | 1 | 0 | 1 | 0 | 7 | Moderate |
| Solomon D  （2022） | 1 | 1 | 1 | 1 | 1 | 0 | 0 | 1 | 0 | 1 | 0 | 7 | Moderate |
| Donne V D (2021) | 1 | 1 | 1 | 0 | 1 | 0 | 0 | 1 | 0 | 0 | 0 | 5 | Moderate |
| Moges N A （2021） | 1 | 1 | 1 | 1 | 1 | 0 | 1 | 1 | 0 | 1 | 0 | 8 | High |
| Garriga C （2020） | 1 | 1 | 1 | 0 | 1 | 0 | 1 | 1 | 1 | 1 | 0 | 8 | High |
| Basha E A （2019） | 1 | 1 | 1 | 0 | 1 | 0 | 1 | 1 | 0 | 1 | 0 | 7 | Moderate |
| Monteiro F （2017） | 1 | 1 | 0 | 1 | 1 | 0 | 1 | 1 | 0 | 1 | 0 | 7 | Moderate |
| Blais M （2015） | 1 | 1 | 1 | 0 | 1 | 0 | 0 | 1 | 0 | 1 | 0 | 6 | Moderate |
| Benoit A C (2014) | 1 | 1 | 1 | 1 | 1 | 0 | 1 | 1 | 1 | 1 | 0 | 9 | High |
| Tesfaye S H （2014） | 1 | 1 | 1 | 1 | 1 | 0 | 0 | 1 | 0 | 1 | 0 | 7 | Moderate |

*A total of 11 items, 1 point for "yes", 0 points for "no" or "unclear", total score of 11 points, 0-3 points for low quality, 4-7 points for moderate quality, 8-11 points for high quality

3.3 NEWCASTLE - OTTAWA QUALITY ASSESSMENT SCALE—COHORT STUDIES

Note: A study can be awarded a maximum of one star for each numbered item within the Selection and Outcome categories. A maximum of two stars can be given for Comparability

**Selection**

1) Representativeness of the exposed cohort (one point)

a) truly representative of the average _______________ (describe) in the community ****

b) somewhat representative of the average ______________ in the community ****

c) selected group of users eg nurses, volunteers

d) no description of the derivation of the cohort

2) Selection of the non exposed cohort (one point)

a) drawn from the same community as the exposed cohort ****

b) drawn from a different source

c) no description of the derivation of the non exposed cohort

3) Ascertainment of exposure (one point)

a) secure record (eg surgical records) ****

b) structured interview ****

c) written self report

d) no description

4) Demonstration that outcome of interest was not present at start of study (one point)

a) yes ****

b) no

**Comparability**

1) Comparability of cohorts on the basis of the design or analysis (two points)

a) study controls for _____________ (select the most important factor) ****

b) study controls for any additional factor **** (This criteria could be modified to indicate specific control for a second important factor.)

**Outcome**

1) Assessment of outcome (one point)

a) independent blind assessment ****

b) record linkage ****

c) self report

d) no description

2) Was follow-up long enough for outcomes to occur (one point)

a) yes (select an adequate follow up period for outcome of interest) ****

b) no

3) Adequacy of follow up of cohorts (one point)

a) complete follow up - all subjects accounted for ****

b) subjects lost to follow up unlikely to introduce bias - small number lost - > ____ % (select an adequate %) follow up, or description provided of those lost) ****

c) follow up rate < ____% (select an adequate %) and no description of those lost

d) no statement

3.4 NEWCASTLE - OTTAWA QUALITY ASSESSMENT SCALE—CASE CONTROL STUDIES

Note: A study can be awarded a maximum of one star for each numbered item within the Selection and Exposure categories. A maximum of two stars can be given for Comparability.

**Selection**

1) Is the case definition adequate?

a) yes, with independent validation ****

b) yes, eg record linkage or based on self reports

c) no description

2) Representativeness of the cases

a) consecutive or obviously representative series of cases ****

b) potential for selection biases or not stated

3) Selection of Controls

a) community controls ****

b) hospital controls

c) no description

4) Definition of Controls

a) no history of disease (endpoint) ****

b) no description of source

**Comparability**

1) Comparability of cases and controls on the basis of the design or analysis

a) study controls for _______________ (Select the most important factor.) ****

b) study controls for any additional factor **** (This criteria could be modified to indicate specific control for a second important factor.)

**Exposure**

1) Ascertainment of exposure

a) secure record (eg surgical records) ****

b) structured interview where blind to case/control status ****

c) interview not blinded to case/control status

d) written self report or medical record only

e) no description

2) Same method of ascertainment for cases and controls

a) yes ****

b) no

3) Non-Response rate

a) same rate for both groups ****

b) non respondents described

c) rate different and no designation

3.5 Scores on all items of the NOS scale* for each cohort study in this research

| **Study** | **Study Type** | **Selection** | | | | **Comparability** | **Outcome/ Exposure** | | | **Score** | **Level** |
| --- | --- | --- | --- | --- | --- | --- | --- | --- | --- | --- | --- |
|  |  | 1) | 2) | 3） | 4） | 5） | 6） | 7） | 8） |  |  |
| Akoko(2024) | cohort study | 1 | 1 | 1 | 0 | 2 | 1 | 1 | 0 | 7 | High |
| Pierce L J(2023) | cohort study | 1 | 1 | 1 | 0 | 2 | 1 | 1 | 1 | 8 | High |
| Brouillette M J(2022) | cohort study | 1 | 0 | 1 | 0 | 2 | 1 | 1 | 0 | 6 | Moderate |
| Bernatsky S(2007) | case control study | 1 | 0 | 1 | 0 | 1 | 1 | 1 | 0 | 5 | Moderate |

*A total of 8 items, divided into three parts: selection (0-4 points), comparability (0-2 points), and outcome (0-3 points). The total score is 9 points. A literature score of 0-4 points is considered low quality, 5-6 points moderate quality, and 7-9 points high quality

Supplementary 4 Certainty of Evidence (GRADE)

4.1 GRADE Evidence Profile for the Prevalence and Associated Factors of PD

| **Outcome** | **N of Studies** | **Relative Effect (95% CI)** | **Certainty of the Evidence (GRADE)** | **Comments** |
| --- | --- | --- | --- | --- |
| Prevalence of PD | 16 (10,204) | 39.0% (28.0 to 52.0) | ⊕〇〇〇 Very Low | ↓ Risk of Bias: Included studies have common methodological limitations. ↓ Inconsistency: Considerable heterogeneity (I² > 95%). ↓ Imprecision: Very wide confidence interval (28% to 52%). |
| Risk Factor: Female Gender | 5 | OR 2.18 (1.73 to 2.75) | ⊕⊕〇〇 Low | ↓ Risk of Bias: 3 high/2 moderate risk studies among 5, indicating risk of bias. ↑ Large Magnitude of Effect: OR > 2. — Inconsistency/Imprecision: Not serious concerns. |
| Protective Factor: Being Married | 4 | OR 0.36 (0.28 to 0.46) | ⊕⊕〇〇 Low | ↓ Risk of Bias: 2 high/2 moderate risk studies among 4, indicating risk of bias. ↑ Large Magnitude of Effect: OR < 0.5. — Inconsistency/Imprecision: Not serious concerns. |
| Risk Factor: Non-disclosure of HIV Status | 4 | OR 4.95 (3.23 to 7.58) | ⊕⊕〇〇 Low | ↓ Risk of Bias: 1 high/3 moderate risk studies among 4, indicating serious risk of bias. ↑ Large Magnitude of Effect: OR > 4. — Inconsistency/Imprecision: Not serious concerns. |
| Risk Factor: Poor ART Adherence | 4 | OR 4.55 (1.96 to 10.54) | ⊕⊕〇〇 Low | — Risk of Bias: 3 high/1 moderate risk studies among 4, quality is acceptable. ↑ Large Magnitude of Effect: OR > 4. ↓ Inconsistency: High heterogeneity (I² = 88.4%). ↓ Imprecision: Very wide confidence interval (1.96 to 10.54). |
| Risk Factor: Low CD4 Count | 3 | OR 2.59 (1.62 to 4.13) | ⊕〇〇〇 Very Low | ↓ Risk of Bias: 2 high/1 moderate risk studies among 3, indicating risk of bias. ↑ Large Magnitude of Effect: OR > 2. — Inconsistency/Imprecision: Not serious concerns (I²=0%, precise CI). ↓ Sparse Data/Indirectness: Only 3 studies, and definition/measurement of exposure may vary. |

* Certainty ratings: All certainty ratings started as 'Low' due to the observational study design. Symbols denote adjustments: ↓ (downgraded), ↑ (upgraded), — (no change).

* Interpretation: High=Very confident; Moderate=Moderately confident; Low=Limited confidence; Very Low=Very little confidence.

Supplementary 5 Sensitivity analysis

5.1 Sensitivity analysis of the prevalence of PD in PLWH by elimination-by-one method.

| **Removed study** | **Pooled prevalence (95%CI)** | **I^2^** |
| --- | --- | --- |
| Akoko (2024) | 40.0% (26.0% to 53.0%) | 96.30% |
| Vondo N (2024) | 40.0% (27.0% to 54.0%) | 96.20% |
| Kesande C (2023) | 39.0% (26.0% to 53.0%) | 96.32% |
| Ma H Q (2023) | 36.0% (25.0% to 48.0%) | 94.51% |
| Pierce L J (2023) | 40.0% (26.0% to 53.0%) | 96.26% |
| Brouillette M J (2022) | 40.0% (26.0% to 53.0%) | 96.65% |
| Solomon D (2022) | 40.0% (26.0% to 54.0%) | 96.24% |
| Donne V D (2021) | 39.0% (25.0% to 53.0%) | 96.50% |
| Moges N A (2021) | 38.0% (25.0% to 52.0%) | 95.98% |
| Garriga C (2020) | 40.0% (26.0% to 53.0%) | 96.22% |
| Basha E A (2019) | 41.0% (29.0% to 54.0%) | 95.57% |
| Monteiro F (2017) | 41.0% (28.0% to 54.0%) | 96.07% |
| Blais M (2015) | 39.0% (26.0% to 53.0%) | 96.49% |
| Benoit A C (2014) | 39.0% (26.0% to 53.0%) | 96.26% |
| Tesfaye S H (2014) | 41.0% (28.0% to 55.0%) | 95.89% |
| Bernatsky S (2007) | 38.0% (25.0% to 52.0%) | 97.52% |
| Overall effect, no study removed | 40.0% (28.0% to 53.0%) | 96.25% |

Supplementary 6 Subgroup Analysis

6.1 Conduct subgroup analysis by era


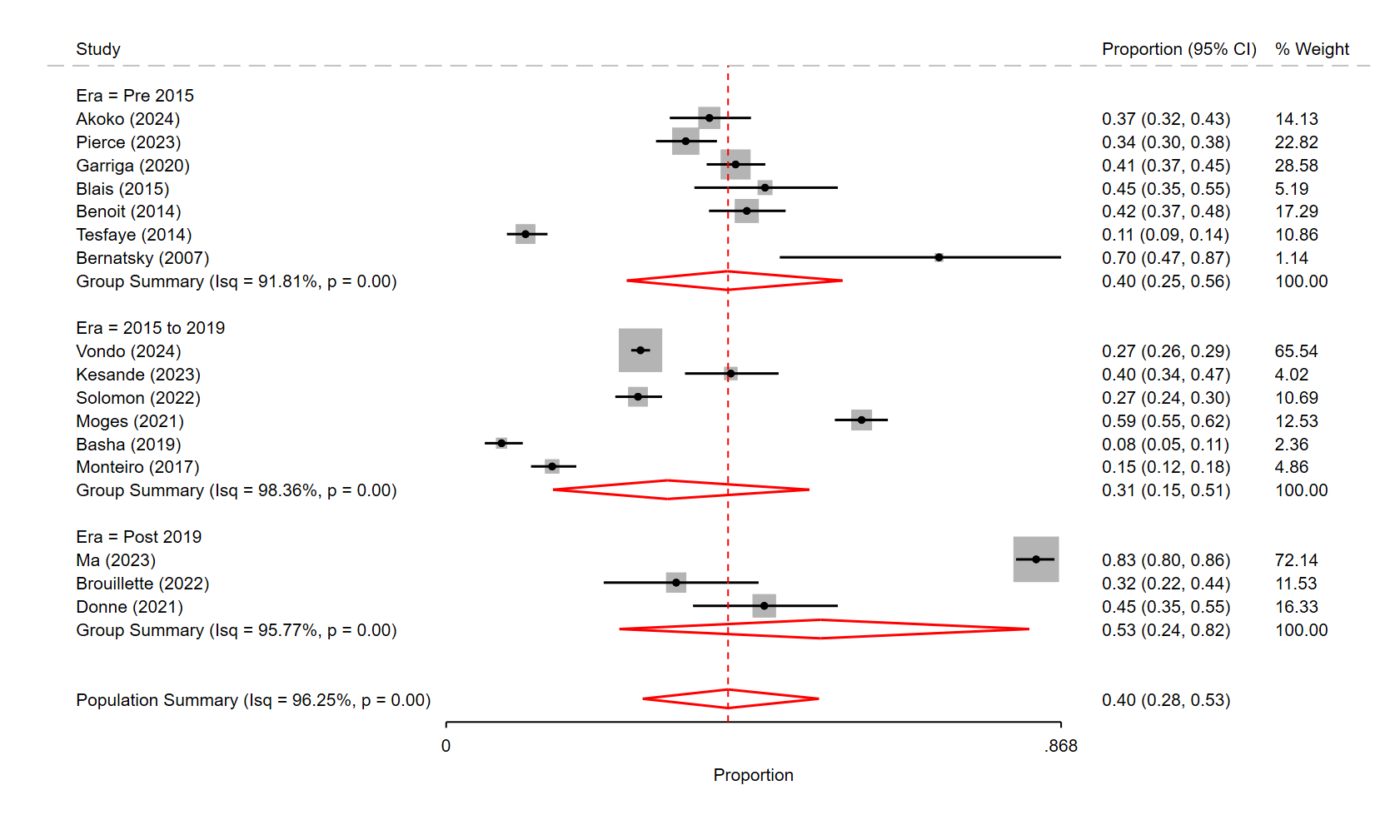


6.2 Conduct subgroup analysis by region


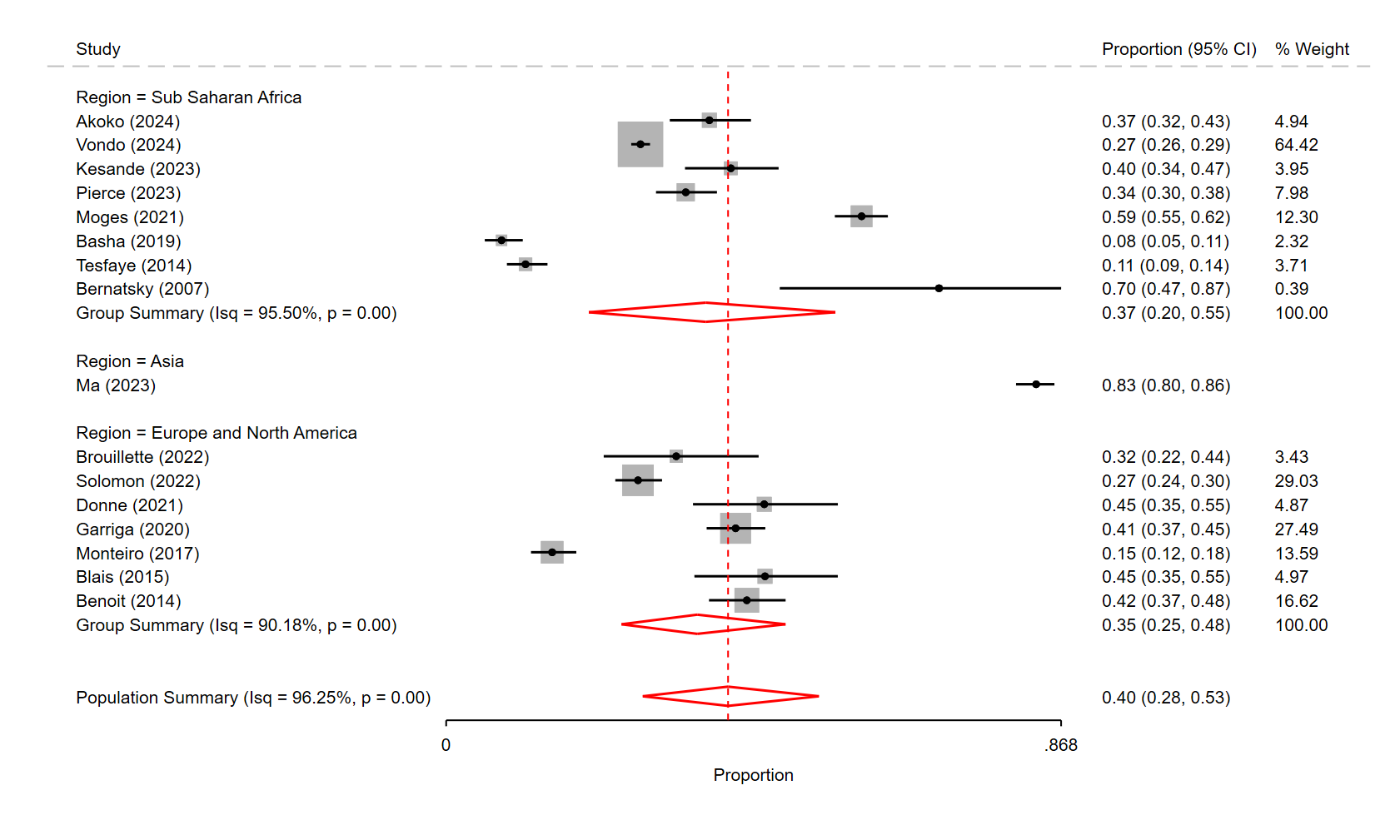


6.3 Conduct subgroup analysis by PD tool

**
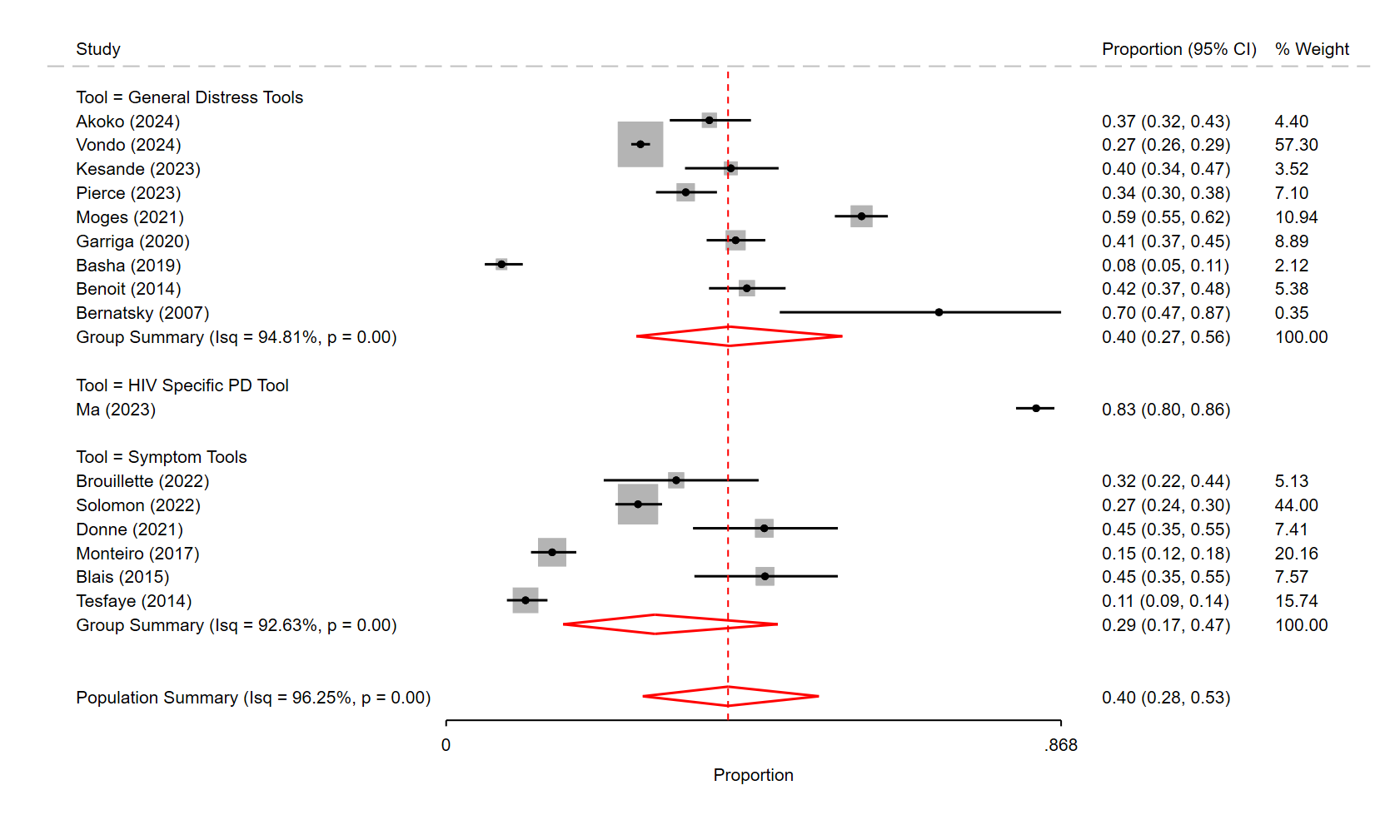
**

6.4 Conduct subgroup analysis by population


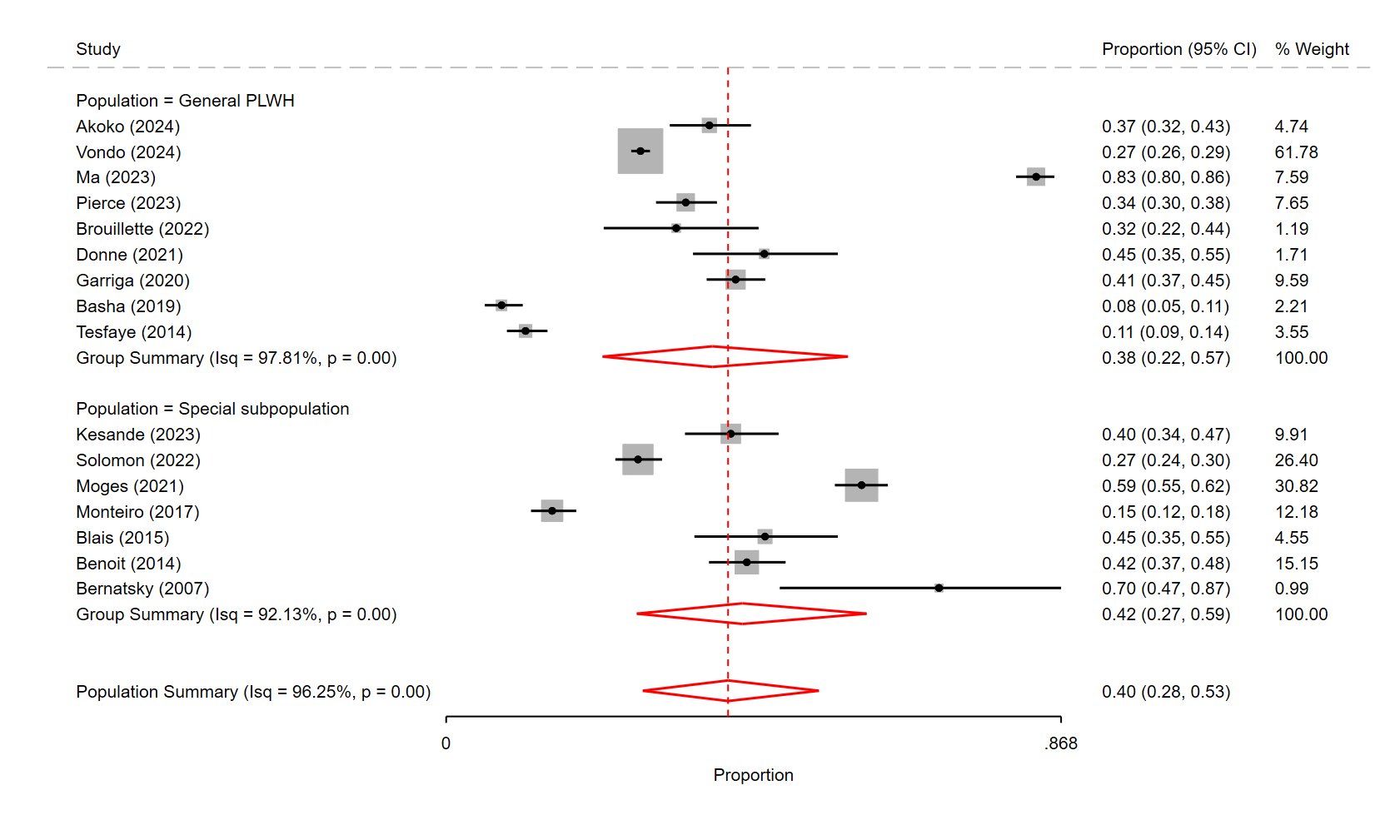


6.5 Conduct subgroup analysis by COVID-19


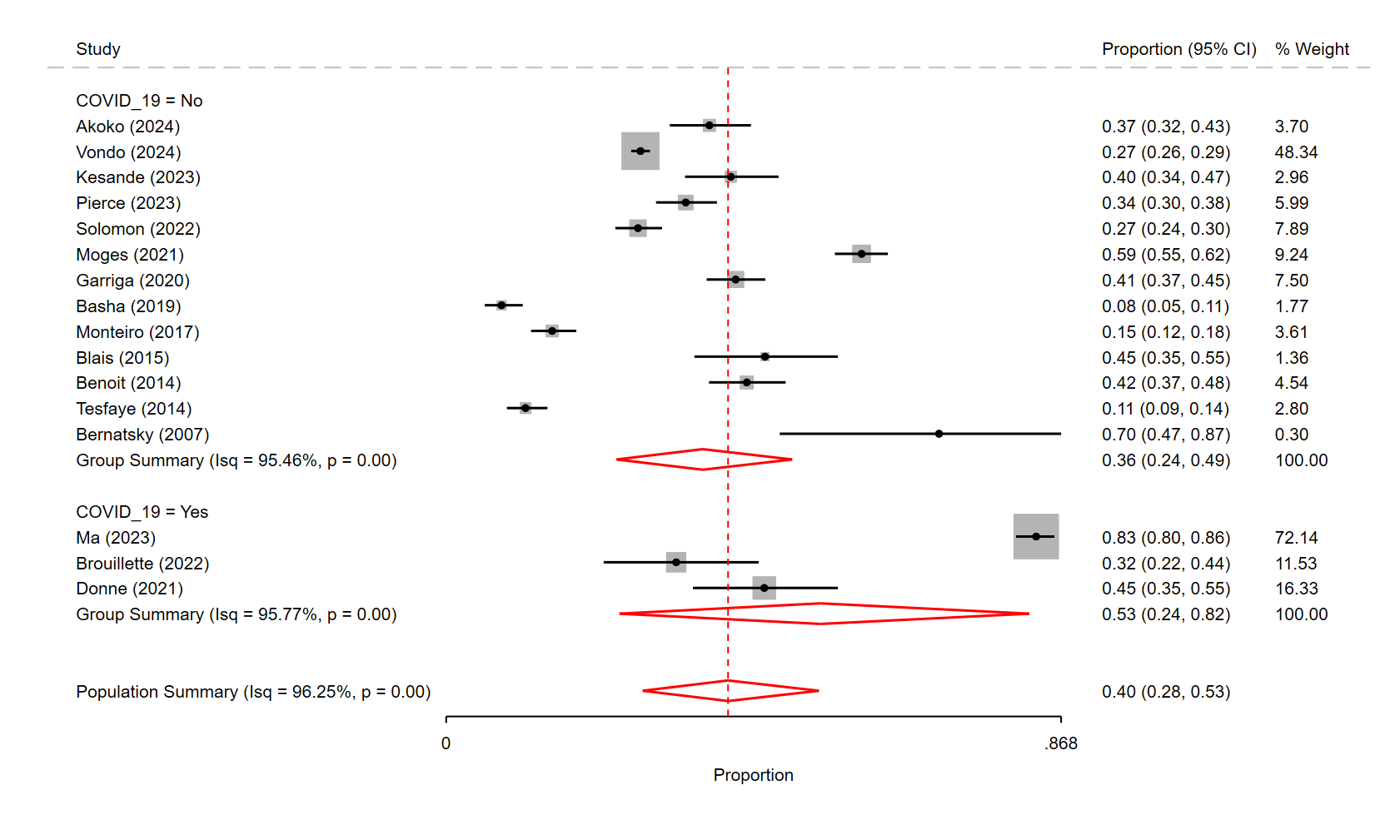


6.6 Conduct subgroup analysis by economic regions


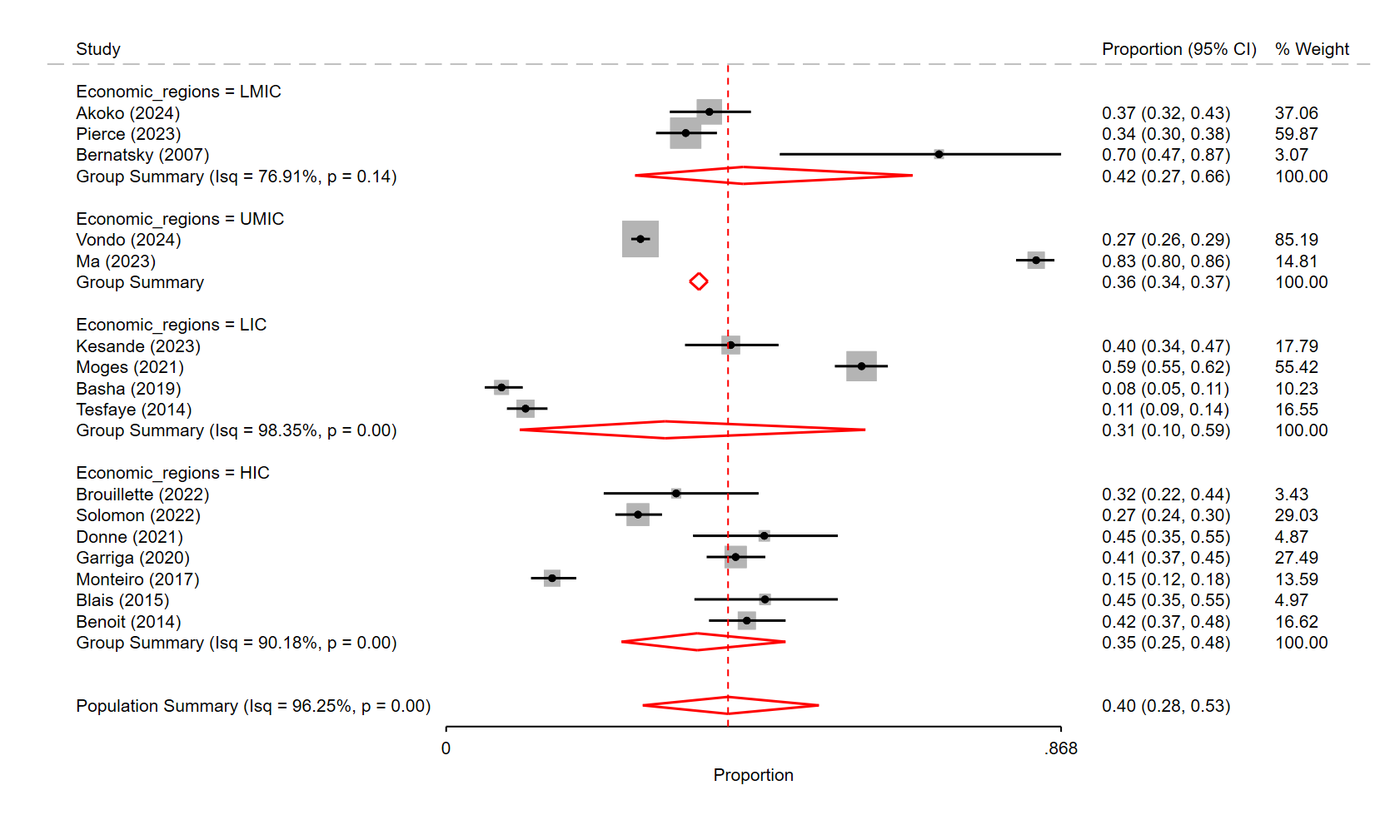


Supplementary 7 Factors Associated with PD

7.1 Age


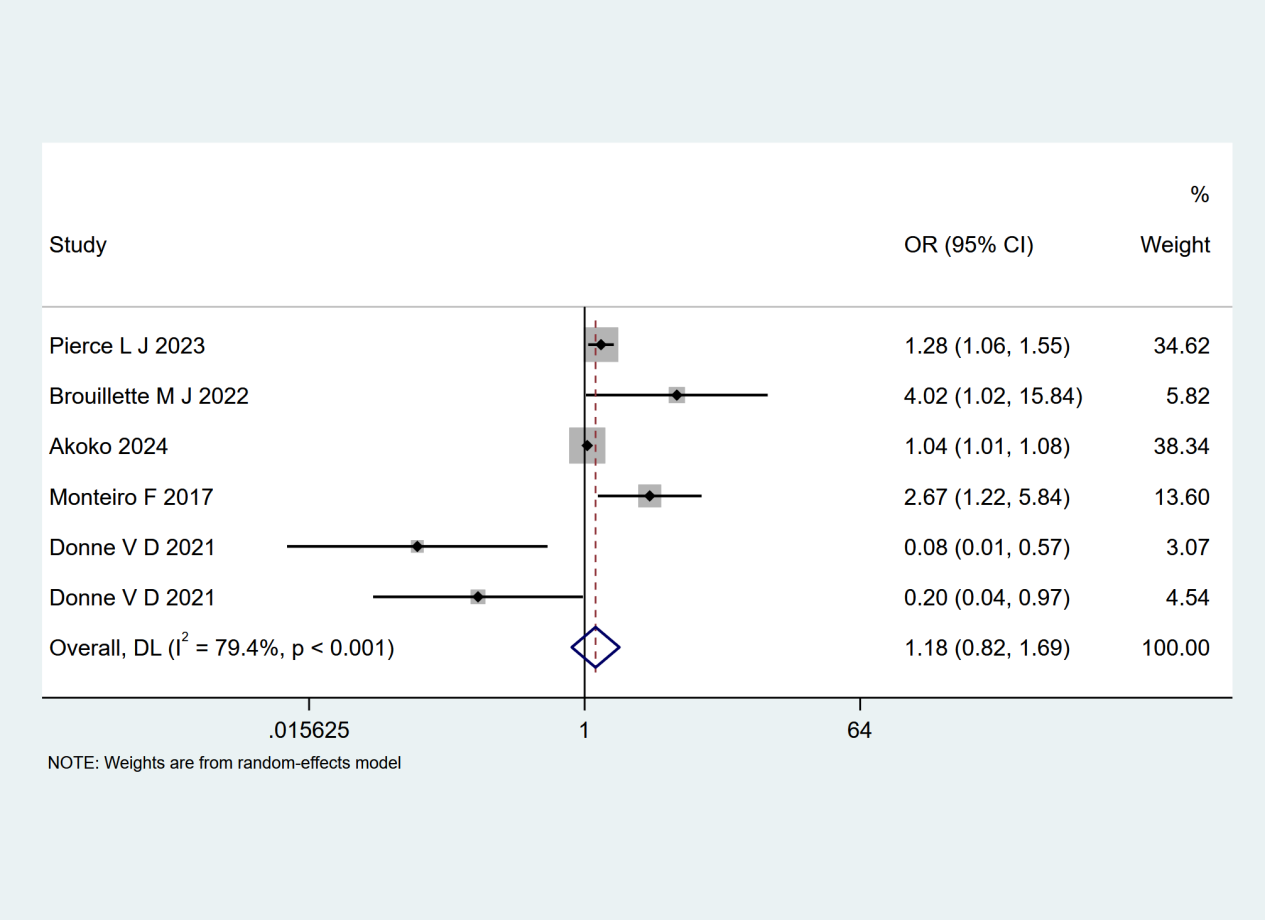


7.2 Gender


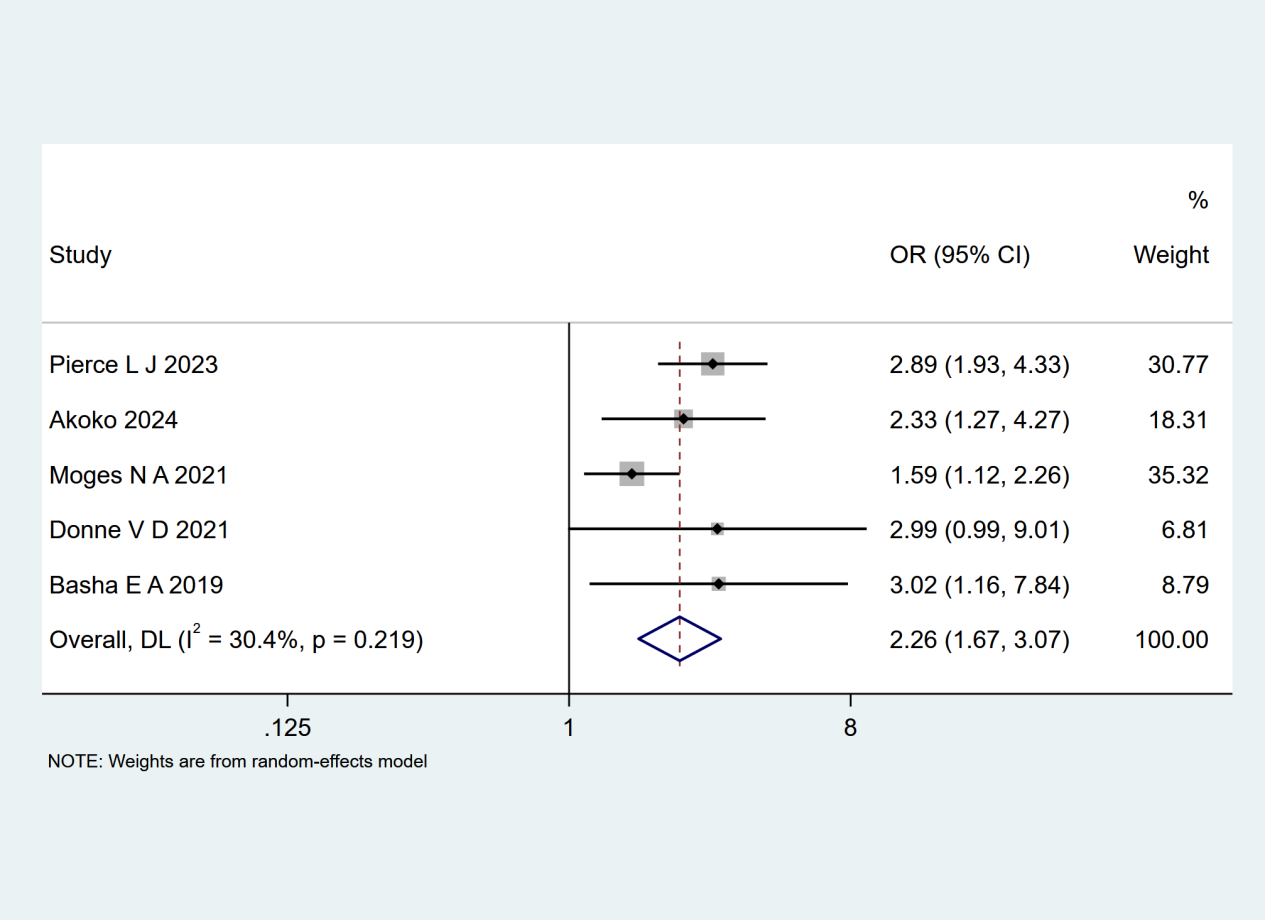


7.3 Marital status


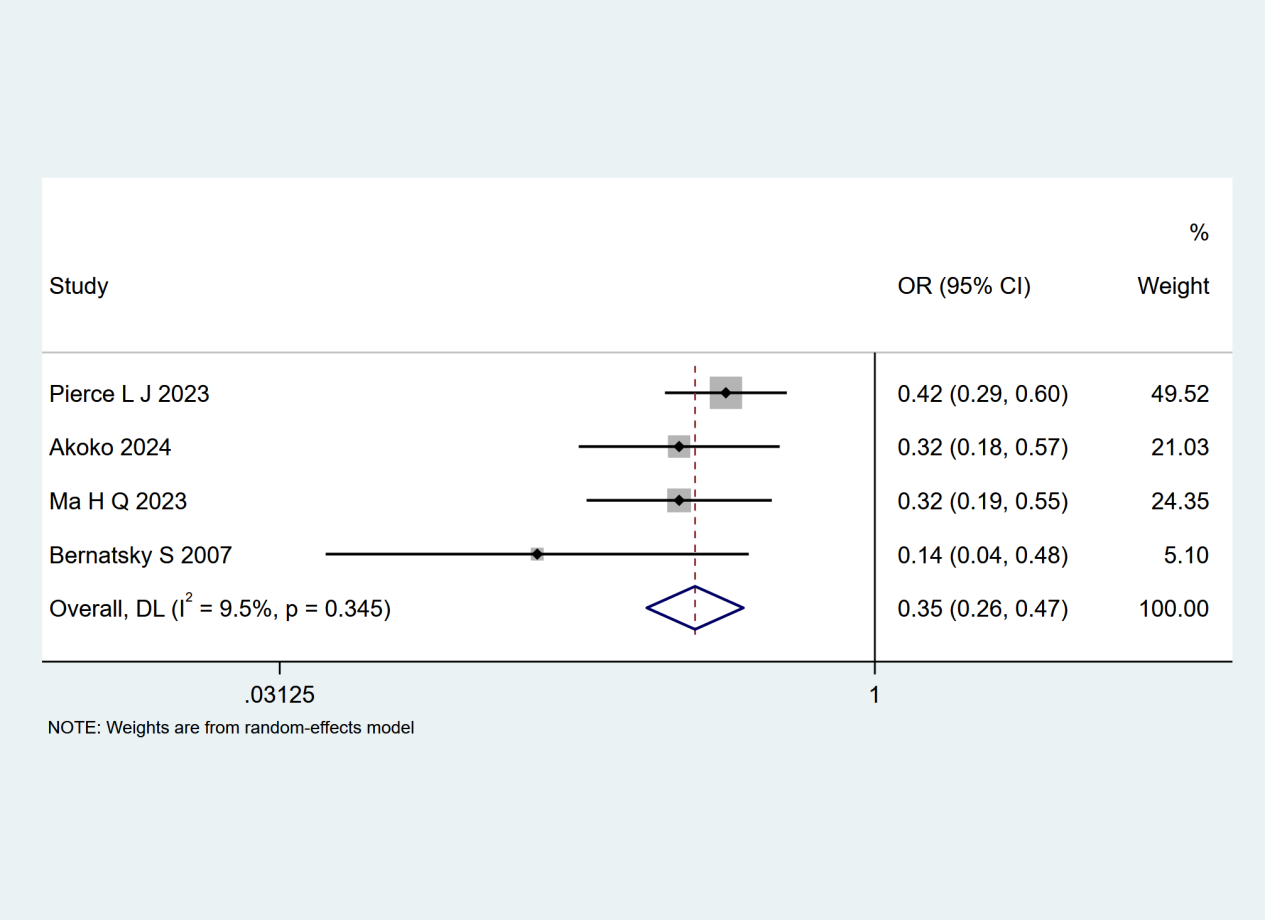


7.4 Current job


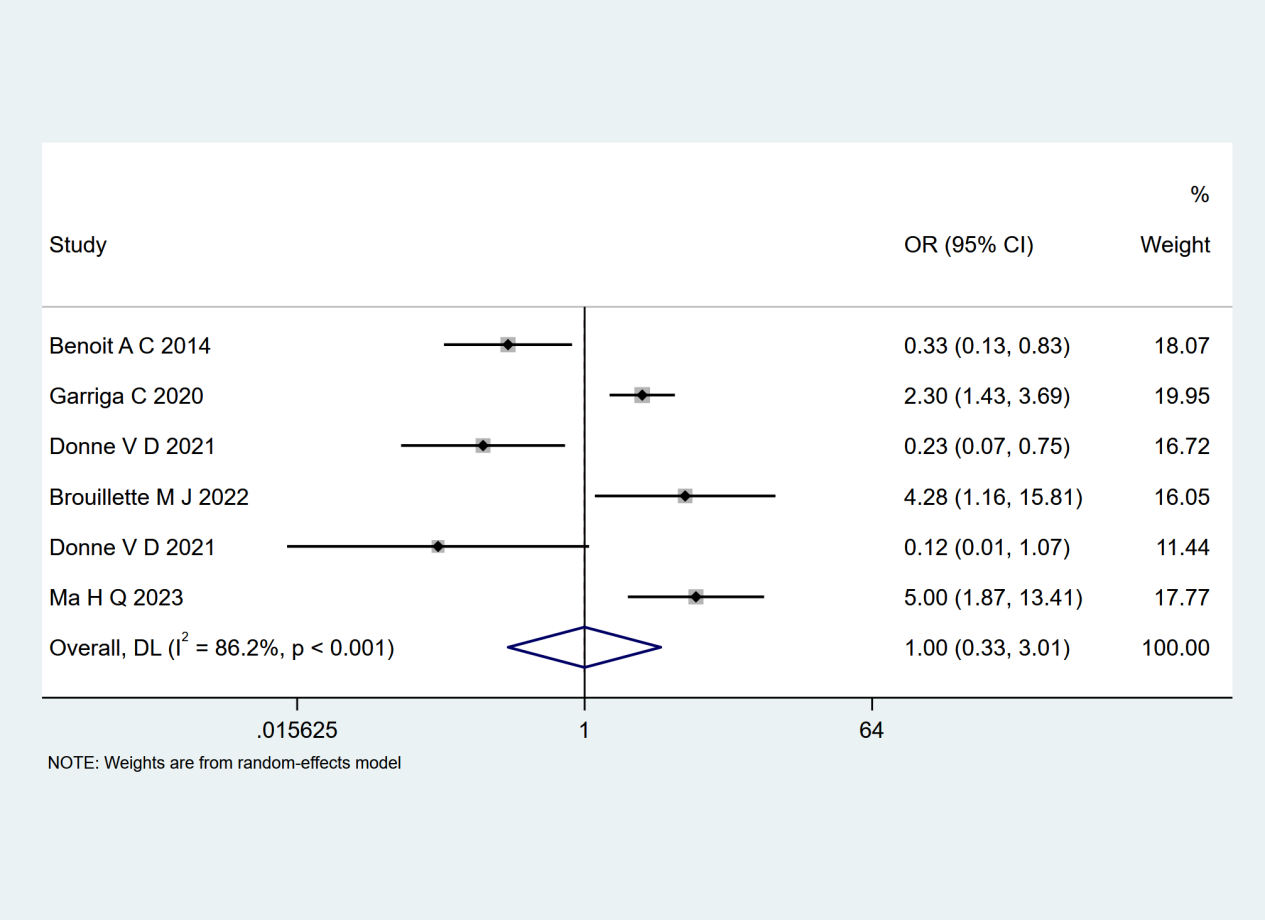


7.5 ART adherence


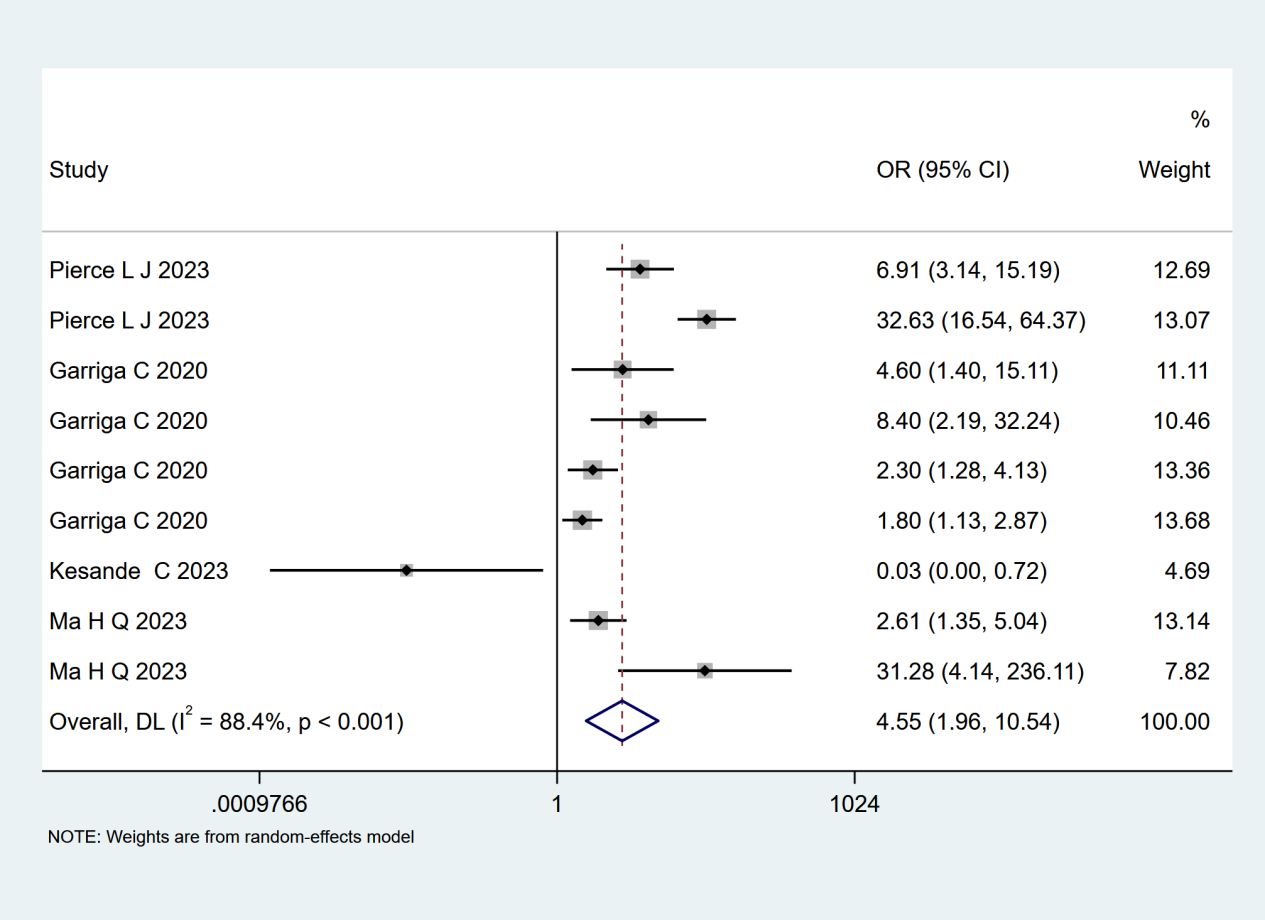


7.6 CD4 count (cells/mm3)


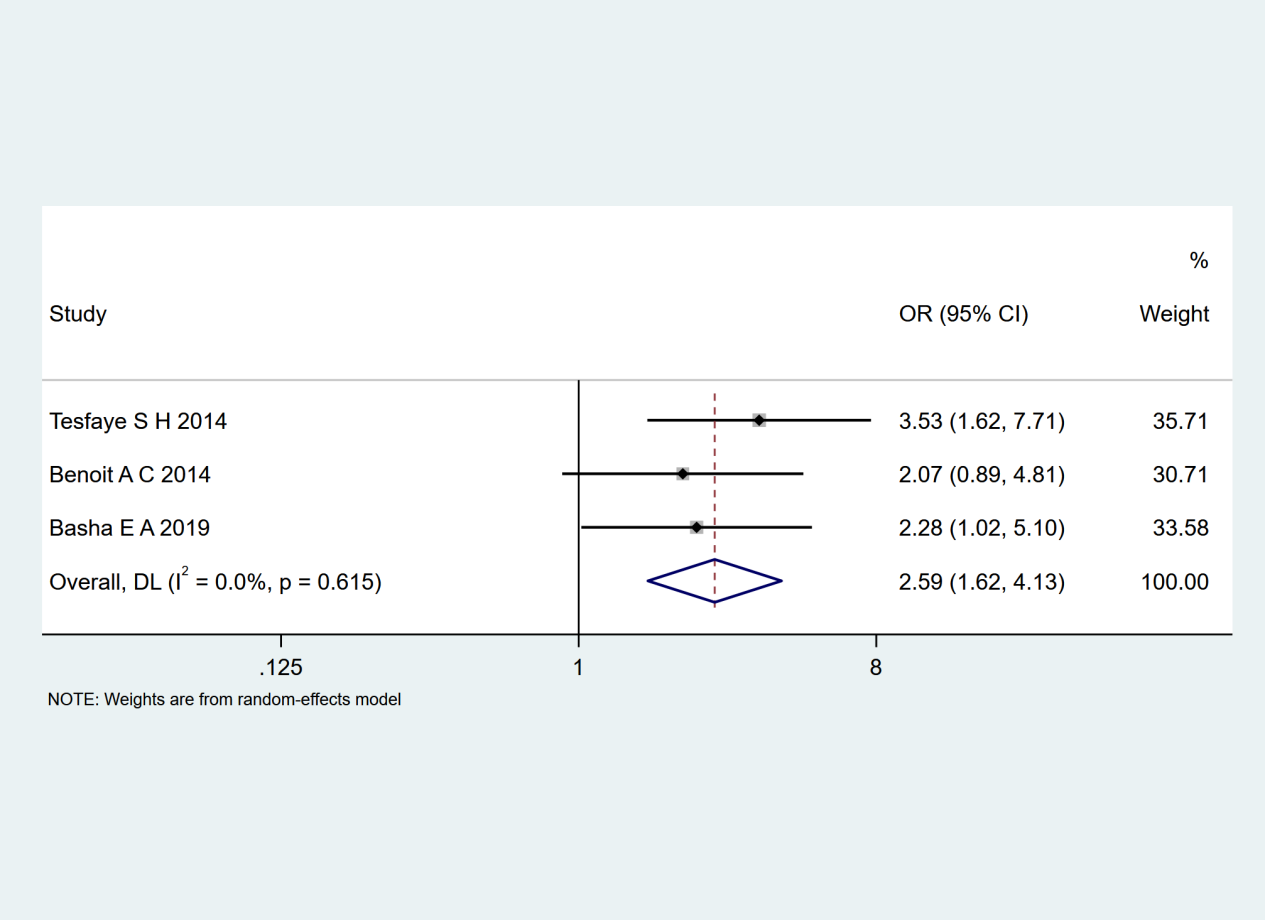


7.7 Education level


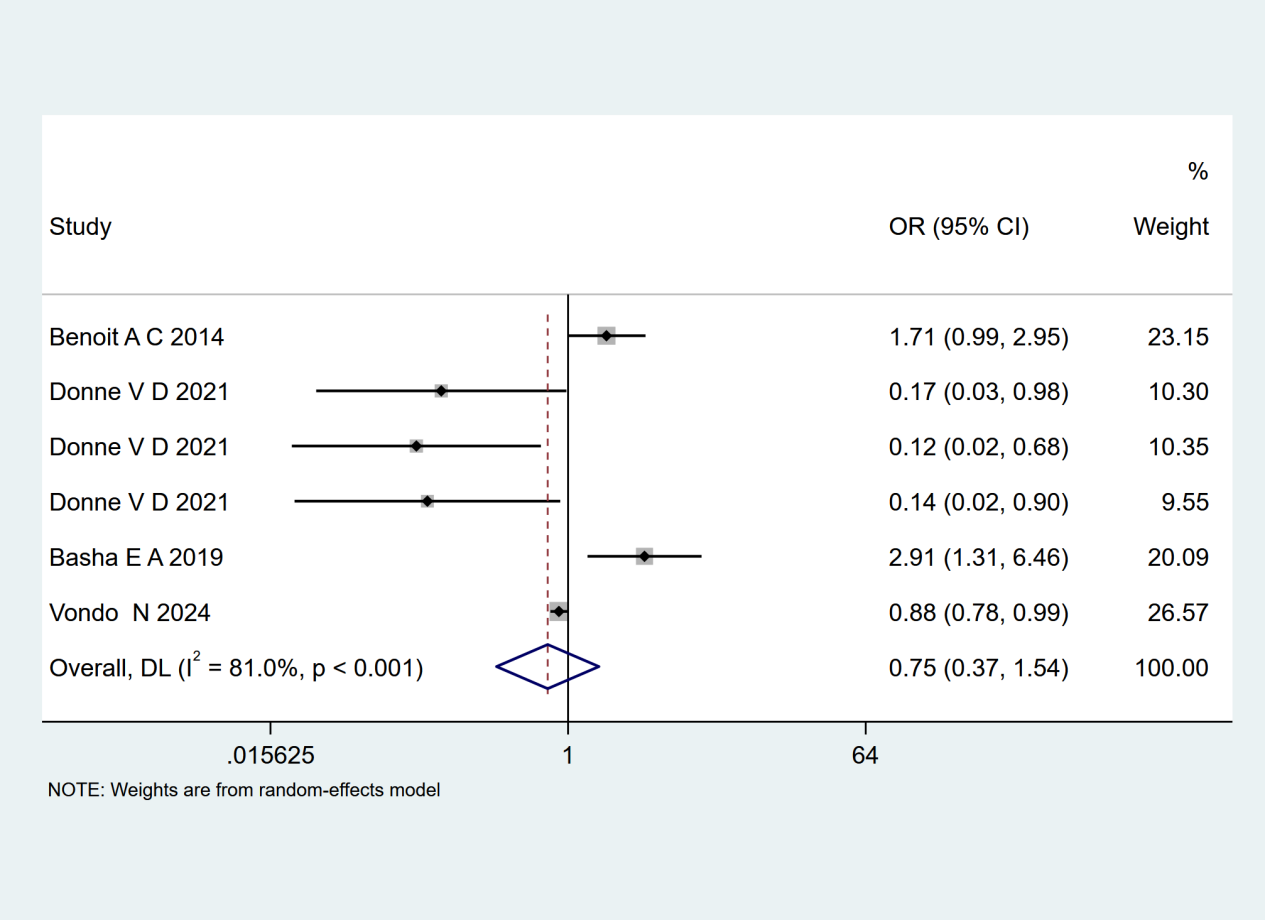


7.8 Disclosure


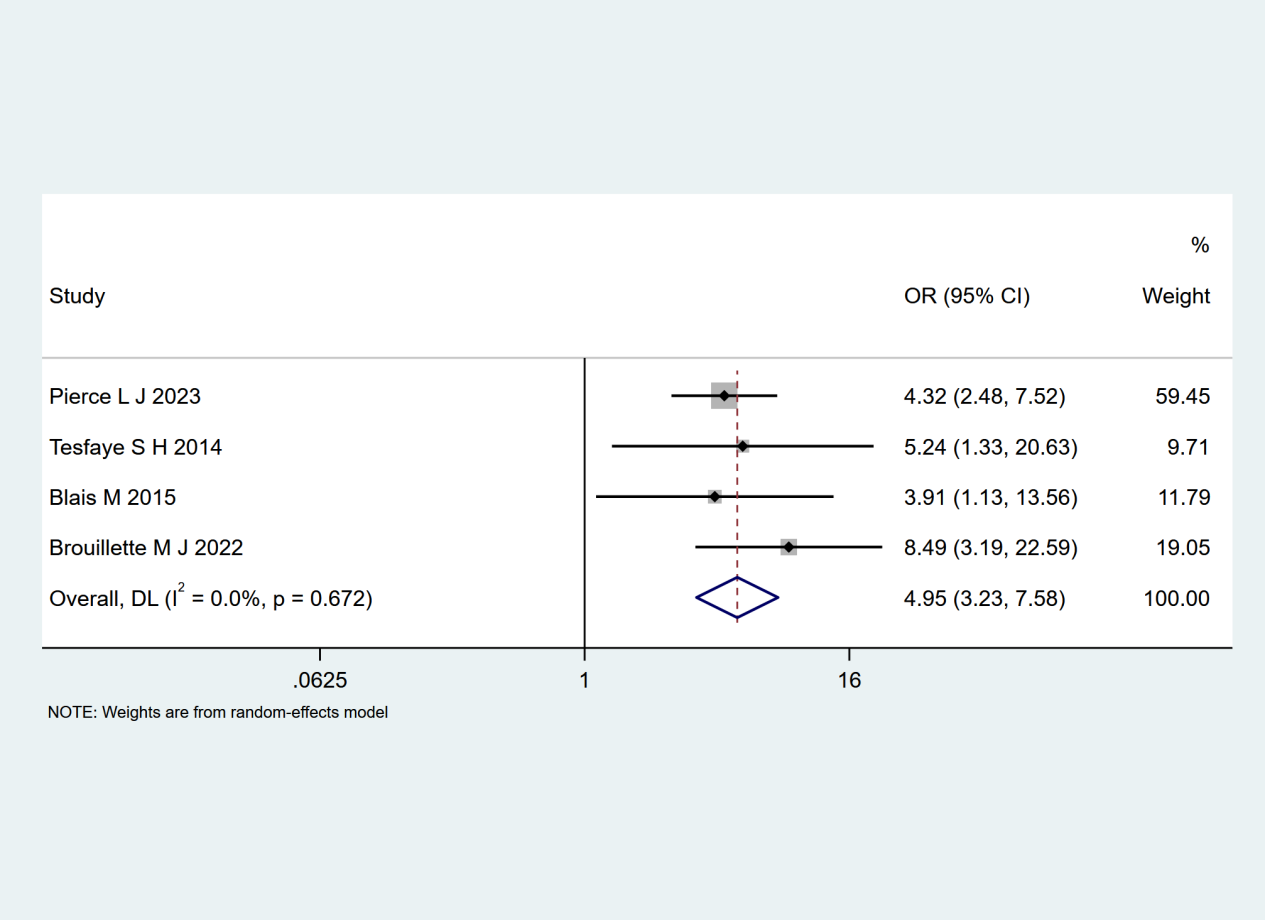


7.9 Self-perceived health


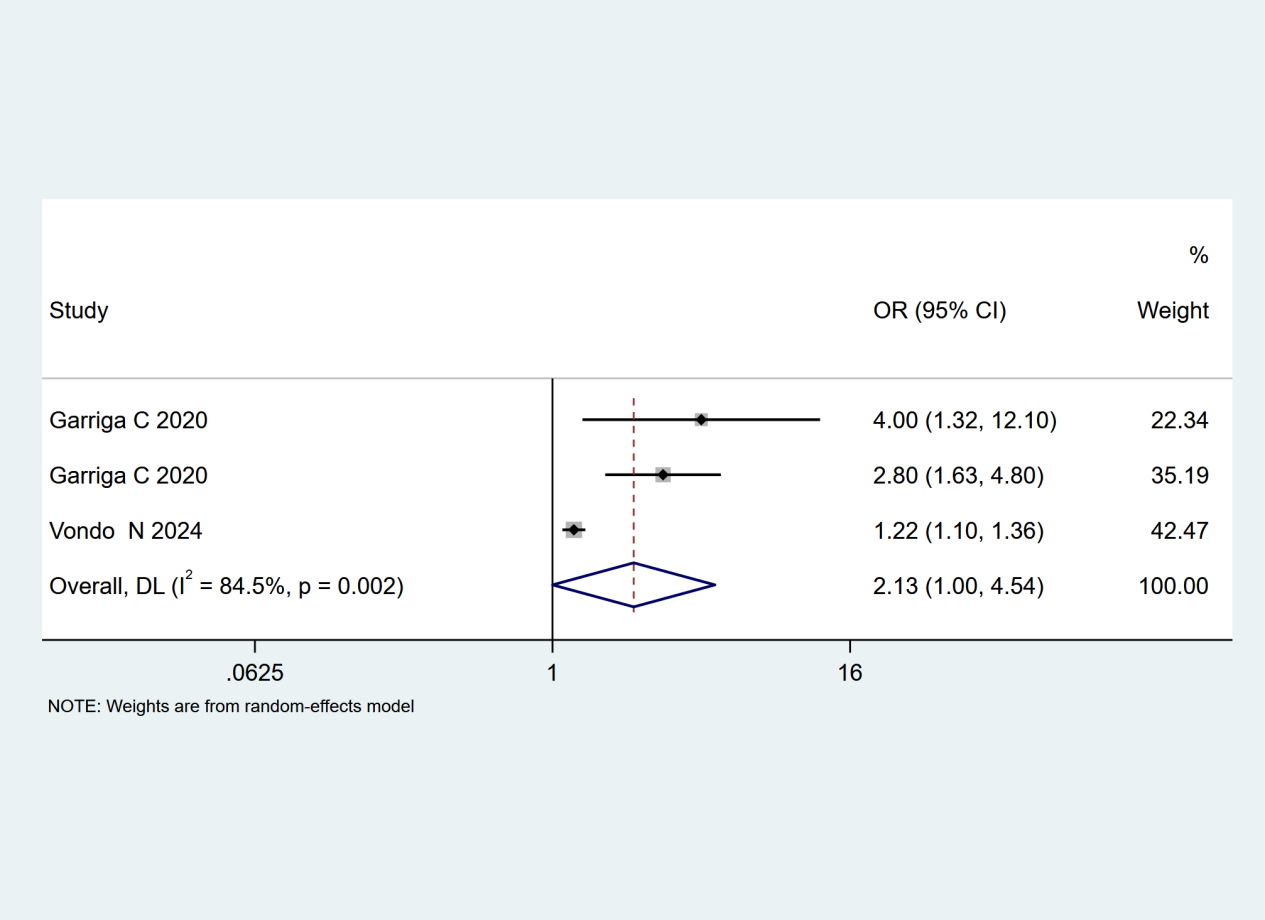

Supplement: Supplementary file 1 [file Supplementary_file_1.docx]
